# Supplementary material for: Circadian regulation of sinoatrial nodal cell pacemaking function: Dissecting the roles of autonomic control, body temperature, and local circadian rhythmicity
Source: PLoS Comput Biol. 2024 Feb 26;20(2):e1011907. doi: 10.1371/journal.pcbi.1011907 (PMC10927146; doi:10.1371/journal.pcbi.1011907)
Supplement: S1 Table — (DOCX) [file pcbi.1011907.s001.docx]

**SUPPORTING INFORMATION**

*Title:* Circadian regulation of sinoatrial nodal cell pacemaking function: dissecting the roles of autonomic control, body temperature, and local circadian rhythmicity

*Pan Li and Jae Kyoung Kim*

Table S1. Model Parameters and Settings

| **Parameter** |  | **Definition** | **Value/setting (reference)** | **Unit** |
| --- | --- | --- | --- | --- |
| A_LCR_ |  | The circadian amplitude of LCR | 0.25(1) | / |
| T_LCR_ |  | The circadian period of LCR | 24(1) | hour |
| CCh_basal_ |  | Basal CCh concentration | 265(2, 3) | nM |
| A_CCh_ |  | The circadian amplitude of CCh | 85(3) | nM |
| T_CCh_ |  | The circadian period of CCh | 24(3) | hour |
| a_cch_ |  | Scaling factor for CCh in aging | 0.25(4) | / |
| k_p_s_ |  | The P-S modulation constant | 450(3, 5) | nM |
| h |  | Hill coefficient | 6(3, 5, 6) | / |
| k_s_p_ |  | The S-P modulation scaling factor | 0.5(3, 6-8) | / |
| BT_a_ |  | The reference BT | 37(9, 10) | °C |
| A_BT_ |  | The circadian amplitude of BT | 1(9, 10) | °C |
| T_BT_ |  | The period of BT variations | 24(9, 10) | hour |
| BT_a,aging_ |  | The reference BT in aged mice | 36.5(10) | °C |
| A_BT, aging_ |  | The circadian amplitude of BT in aged mice | 0.5(10) | °C |
| a_cat_ |  | Scaling factor for I_CaT_ in aging | 0.46(11) | / |
| a_cal_ |  | Scaling factor for I_CaL_ in aging | 0.69(11) | / |
| a_hcn_ |  | Scaling factor for I_HCN_ in aging | 0.48(11) | / |
| v_hcn_ |  | Activation shift of I_HCN_ in aging | 12(11) | mV |
| a_nak_ |  | Scaling factor for I_NaK_ in aging | 1.5(12) | / |
| a_ncx_ |  | Scaling factor for I_NCX_ in aging | 0.5(13) | / |
| a_kr_ |  | Scaling factor for I_Kr_ in aging | 0.92(12) | / |
| a_ks_ |  | Scaling factor for I_Ks_ in aging | 1.6(12) | / |
| a_up_ |  | Scaling factor for J_up_ in aging | 0.7(13) | / |
| a_rel_ |  | Scaling factor for J_Rel_ in aging | 0.2(13) | / |
| a_kmf_ |  | Scaling factor for K_mf_ in aging | 1.25(13) | / |
| B |  | Scaling factor for the effects of CRBT on ion channels | 0.008(14, 15) | K^-1^ |
| B_SNA_ |  | Scaling factor for the effects of CRBT on SNA | 0.096(16) | K^-1^ |
| B_PNA_ |  | Scaling factor for the effects of CRBT on PNA | -0.04(16) | K^-1^ |
| Q_10_ |  | The temperature coefficient | 2.18 for *I_to_* and *I_sus_* (17, 18)  1.7 for *I_Na_* (17)  2.3 for *I_CaL_*(17)  1.5 for *I_CaT_* (17)  1.5 for *I_Kr_*, *I_Ks_* and *I_KACh_* (15)  2.0 for *I_HCN_* (18, 19)  2.6 for *J_up_* (20)  1.6 for *I_NCX_* (20, 21)  2.1 for *I_NaK_* (22)  1.25 for *J_Rel_* (23)  1.3 for *J_diff_* and *J_tr_* (23)  1.6 for Ca^2+^ buffers (24) | / |
| e_cch_ |  | Standalone effects of CCh | I_KACh_ activation (2) | |
| e_iso_ |  | Standalone effects of ISO | 15.9% increase in *G_st_* (2, 3, 25) | |
|  |  |  | 15.9% increase in *G_Na1.1_* (2, 3, 25) | |
|  |  |  | 85.5% increase in *G_CaT_* (2, 3, 25) | |
|  |  |  | 112% increase in *G_CaL_* (2, 3, 25) | |
|  |  |  | 15.9% increase in *G_K1_* (2, 3, 25) | |
|  |  |  | 15.9% increase in *G_Ks_* (2, 3, 25) | |
|  |  |  | 15.9% increase in *G_to_* (2, 3, 25) | |
|  |  |  | 112% increase in *V_Rel_* (2, 3, 25) | |
|  |  |  | 52.83% decrease in *k_up_* (2, 3, 25) | |
|  |  |  | Altered steady-state activation (*V_HCN_* = 87.618mV) and gating properties in *I_HCN_* (2, 3, 25) | |
|  |  |  | 16.6% increase in *G_Kr_* and 5.3 mV negative shift of activation (2, 3, 25) | |

Supplemental References

1. D’souza A, Wang Y, Anderson C, Bucchi A, Baruscotti M, Olieslagers S, et al. A circadian clock in the sinus node mediates day-night rhythms in Hcn4 and heart rate. Heart Rhythm. 2021;18(5):801-10.

2. Ding Y, Lang D, Yan J, Bu H, Li H, Jiao K, et al. A phenotype-based forward genetic screen identifies Dnajb6 as a sick sinus syndrome gene. Elife. 2022;11:e77327.

3. Barazi N, Polidovitch N, Debi R, Yakobov S, Lakin R, Backx PH. Dissecting the roles of the autonomic nervous system and physical activity on circadian heart rate fluctuations in mice. Frontiers in physiology. 2021;12:692247.

4. Freeling JL, Li Y. Age-related attenuation of parasympathetic control of the heart in mice. International journal of physiology, pathophysiology and pharmacology. 2015;7(3):126.

5. Behar J, Ganesan A, Zhang J, Yaniv Y. The autonomic nervous system regulates the heart rate through cAMP-PKA dependent and independent coupled-clock pacemaker cell mechanisms. Frontiers in physiology. 2016;7:419.

6. Levy MN. Brief reviews: sympathetic-parasympathetic interactions in the heart. Circulation research. 1971;29(5):437-45.

7. van Koppen CJ, Kaiser B. Regulation of muscarinic acetylcholine receptor signaling. Pharmacology & therapeutics. 2003;98(2):197-220.

8. Lee N, Fraser C. Cross-talk between m1 muscarinic acetylcholine and beta 2-adrenergic receptors. cAMP and the third intracellular loop of m1 muscarinic receptors confer heterologous regulation. Journal of Biological Chemistry. 1993;268(11):7949-57.

9. Beaudry JL, McClelland GB. Thermogenesis in CD-1 mice after combined chronic hypoxia and cold acclimation. Comparative Biochemistry and Physiology Part B: Biochemistry and Molecular Biology. 2010;157(3):301-9.

10. Sanchez-Alavez M, Alboni S, Conti B. Sex-and age-specific differences in core body temperature of C57Bl/6 mice. Age. 2011;33:89-99.

11. Larson ED, St. Clair JR, Sumner WA, Bannister RA, Proenza C. Depressed pacemaker activity of sinoatrial node myocytes contributes to the age-dependent decline in maximum heart rate. Proceedings of the National Academy of Sciences. 2013;110(44):18011-6.

12. Tellez JO, Mączewski M, Yanni J, Sutyagin P, Mackiewicz U, Atkinson A, et al. Ageing‐dependent remodelling of ion channel and Ca2+ clock genes underlying sino‐atrial node pacemaking. Experimental physiology. 2011;96(11):1163-78.

13. Liu J, Sirenko S, Juhaszova M, Sollott SJ, Shukla S, Yaniv Y, et al. Age-associated abnormalities of intrinsic automaticity of sinoatrial nodal cells are linked to deficient cAMP-PKA-Ca2+ signaling. American Journal of Physiology-Heart and Circulatory Physiology. 2014;306(10):H1385-H97.

14. Collet A, Bragard J, Dauby P. Temperature, geometry, and bifurcations in the numerical modeling of the cardiac mechano-electric feedback. Chaos: An Interdisciplinary Journal of Nonlinear Science. 2017;27(9).

15. Fenton FH, Gizzi A, Cherubini C, Pomella N, Filippi S. Role of temperature on nonlinear cardiac dynamics. Physical Review E. 2013;87(4):042717.

16. Walsh R. Heart rate and its neural regulation with rising body temperature in anesthetized rats. American Journal of Physiology-Legacy Content. 1969;217(4):1139-43.

17. Zhang H, Holden A, Kodama I, Honjo H, Lei M, Varghese T, et al. Mathematical models of action potentials in the periphery and center of the rabbit sinoatrial node. American Journal of Physiology-Heart and Circulatory Physiology. 2000;279(1):H397-H421.

18. Kurata Y, Hisatome I, Imanishi S, Shibamoto T. Dynamical description of sinoatrial node pacemaking: improved mathematical model for primary pacemaker cell. American Journal of Physiology-Heart and Circulatory Physiology. 2002;283(5):H2074-H101.

19. Wu Y, Wang Q, Granger J, Reyes Gaido OE, Aguilar EN, Ludwig A, et al. HCN channels sense temperature and determine heart rate responses to heat. bioRxiv. 2023:2023.09. 02.556046.

20. Puglisi J, Bassani R, Bassani J, Amin J, Bers D. Temperature and relative contributions of Ca transport systems in cardiac myocyte relaxation. American Journal of Physiology-Heart and Circulatory Physiology. 1996;270(5):H1772-H8.

21. Marengo FD, Wang S-y, Langer GA. The effects of temperature upon calcium exchange in intact cultured cardiac myocytes. Cell Calcium. 1997;21(4):263-73.

22. Sakai R, Hagiwara N, Matsuda N, Kassanuki H, Hosoda S. Sodium‐‐potassium pump current in rabbit sino‐atrial node cells. The Journal of Physiology. 1996;490(1):51-62.

23. Fu Y, Zhang G-Q, Hao X-M, Wu C-H, Chai Z, Wang S-Q. Temperature dependence and thermodynamic properties of Ca2+ sparks in rat cardiomyocytes. Biophysical journal. 2005;89(4):2533-41.

24. Lindblad D, Murphey C, Clark J, Giles W. A model of the action potential and underlying membrane currents in a rabbit atrial cell. American Journal of Physiology-Heart and Circulatory Physiology. 1996;271(4):H1666-H96.

25. Kharche S, Yu J, Lei M, Zhang H. A mathematical model of action potentials of mouse sinoatrial node cells with molecular bases. American Journal of Physiology-Heart and circulatory physiology. 2011;301(3):H945-H63.
