# Supplementary material for: Circadian regulation of sinoatrial nodal cell pacemaking function: Dissecting the roles of autonomic control, body temperature, and local circadian rhythmicity
Source: PLoS Comput Biol. 2024 Feb 26;20(2):e1011907. doi: 10.1371/journal.pcbi.1011907 (PMC10927146; doi:10.1371/journal.pcbi.1011907)
Supplement: Attachment — Submitted filename: PLoSCB-Point-by-Point-Response.docx [file pcbi.1011907.s002.docx]

The submitted manuscript utilized mathematical modeling to simulate cardiac pacemaking controlled by central and peripheral clocks uncovering potential impacts of aging in circadian regulation of heart rate. The reviewers acknowledge that synergistic interactions between PNA, SNA, and LCR regulating cardiac pacemaking are interesting. However, reviewers raised some major concerns including **lack of technical details, potential effects of temperature, and impact of calcium signaling over aging**. Importantly, it will be critical to **clearly define the terms used in the manuscript** improving clarity of conclusions and overall readability of the manuscript.

We thank the editor and all reviewers for your insightful comments and kind suggestions. In response, we have extensively revised the manuscript to provide full technical details in our revised methods section regarding model development, validation, and simulation protocols, with a new Table S1 (see Supporting Information) to list all parameter definitions and settings; extend our model to incorporate the circadian rhythm of body temperature in both adult and aged mice; extend our aging model to account for a more comprehensive description of ion channel remodeling, altered Calcium signaling, parasympathetic nervous activities (PNA) and circadian rhythms of body temperature (CRBT) disruption in aged mice. In addition, to improve the overall readability of the manuscript, we have also extensively reduced the number of non-standard abbreviations used in our manuscript; included a new Table 1 to list the glossary; revised our model schematic (Fig 1) to include a list of key term definitions; and included a new Table 2 immediately following Fig 1 to list definitions of non-standard abbreviations.

Below, we give our detailed responses to the reviewers’ comments and describe the changes in our manuscript. Our responses appear in blue. The revised portions of the manuscript have been reported in each response, marked in italic font. The revised portion is represented as blue text in the main text and supplementary materials.

***Reviewers’ Comments:***

**Reviewer #1:**

Summary: This report features a mathematical model describing autonomic control and LCR in sinoatrial nodal cells that attempts to capture distinct circadian patterns in adult and aged mice. Although the paper is interesting, a number of technical concerns require clarification, and additional modeling of how calcium oscillations change with aging is required.

**Major Comments**

1. The entire paper is about how “PNA, SNA, and LCR, … synergistically work together”. By definition, “synergistically” means that together they achieve more than in isolation. Please be specific and refer to your results for separate or concerted actions and clearly formulate (and prove) conclusions as claimed in your title. How much synergy (in quantitative terms) is achieved in your model? Please add this data in the abstract and provide clear conclusions after or within the discussion section.

RE: We agree with you that “synergy” by definition means that together they achieve more than in isolation, and in our study, “synergy” is applied to our results regarding flexibility, not performance or robustness. In the revised manuscript, our results were extended mostly to highlight the distinct roles of ANS, LCR, and circadian rhythms in body temperature (CRBT), and their alliance in optimizing SANC pacemaking function. To address this issue and improve clarity, we removed the use of “synergy” or “synergistically” in our manuscript title, abstract, figures, and results to more accurately summarize our key findings.

Page 1, title: “*Circadian regulation of sinoatrial nodal cell pacemaking function: dissecting the roles of autonomic control, body temperature, and local circadian rhythmicity*”

Page 2, abstract: “Our model underscores *how the alliance among ANS, CRBT, and LCR* achieves circadian flexibility to cover a wide range of firing rates in SANC, performance to achieve maximal firing rates, while preserving robustness to generate rhythmic firing patterns irrespective of external conditions*.”*

Page 2, abstract: “Our work *sheds* light on the *critical alliance of ANS, CRBT, and LCR* in regulating time-of-day cardiac pacemaking function and dysfunction, offering insights into novel therapeutic targets for the prevention and treatment of cardiac arrhythmias.”

Page 3, author summary: “Our results unveiled distinct roles played by these clocks in determining circadian patterns of sinoatrial nodal cells, *shedding light on their critical alliance* in regulating time-of-day cardiac pacemaking function and dysfunction.”

Page 13, results: “*The alliance of ANS, CRBT, and LCR* is essential in optimizing the time-of-day SANC function in adult mice”

Page 16, results: “Fig 3. The *alliance* of *ANS, CRBT and* LCR is essential to *achieve circadian flexibility and performance while preserving robustness* in SANC automaticity …… ”

Page 21, discussion: “However, under aging conditions, *the cooperative alliance of ANS, CRBT, and LCR* is disrupted, resulting in a substantial reduction in the performance and flexibility of SANC.....”

2. In fig3C, I see only a synergistic effect for flexibility, while the title claims synergistic effects also for robustness and performance. Please explain.

RE: Indeed, we agree that in our study, “synergy” is applied to our results regarding flexibility, not performance or robustness. To address this issue, we have revised our manuscript title and the legend of Fig 3 accordingly (see our response to comment 1).

3. In Methods you refer to three papers (27, 31, 40) for your model. You also say that “In this study, we present a novel mathematical model”. In any case, please be more specific and cite the closest oldest model published previously that was used as a basis for your novel model. Also, describe in full detail what is novel compared to previous models and, very importantly, please provide full computer code for your new model for all of your simulations. Your working code should be in the paper supplement or in GitHub or any other public database. This will allow anyone (incl. reviewers, editors, and readers) to reproduce your results and to use in further studies by running your actual (tested and working) code.

RE: To address this issue, we: (1) revised the methods section to be more specific, and cite the closest and oldest model published previously as our baseline model in this study; (2) provided in full detail our original contribution modeling circadian regulation of SANC pacemaking in mice; (3) will upload model codes underlying our study to our group’s website on GitHub for public download once the manuscript is accepted.

Page 7, introduction: “In this study, we present a novel mathematical model that captures the intricate regulation of SANC by *ANS, CRBT, and LCR* in mice (Fig 1) *(Table 2)*. The model accurately reproduces diverse circadian patterns as shown in previous experimental studies in adult and aged mice (19, 25, 38). Utilizing the model, we elucidate the specific roles of *ANS, CRBT, and LCR in attaining circadian flexibility, performance, and robustness in SANC*.”

Page 20, discussion: “*Our study sheds light on a critical yet understudied dimension of time-of-day interactions between master and local clocks in cardiac pacemaking function and dysfunction*, by developing a model of *ANS, CRBT, and LCR* in SANC to recapitulate diverse circadian patterns in both adult and aged mice.”

Page 24, methods: “*Autonomic, electrophysiologic, and circadian properties of cardiac function are species-dependent (26, 79). Given the availability of experimental data in mice, our model is constructed to be mouse-specific. We used the mathematical model of mouse SANC AP developed by Ding et al. (48, 51), based on the original work of Kharche et al. (75), as our baseline model. All baseline model definitions, equations, and parameter settings were unchanged from those previously described and implemented by Ding et al. (48). Due to the absence of circadian regulation in the baseline model, we extended it to quantitatively describe circadian variations in ANS, CRBT, and LCR for both adult and aged mice. Detailed descriptions of model equations are provided in the following section, along with all parameter values and settings listed in Table S1 (Supporting Information).”*

Page 30, methods: “*All model simulations were performed with parallel computing on a ThinkStation P620 tower workstation with an AMD Threadripper processor. Model codes were implemented in MATLAB (Version: 9.13.0 (R2022b)) using ode15s, and will be provided for public download from https://github.com/Mathbiomed/CircSANC.*”

4. You wrote: “This modeling choice aligns with earlier experimental findings on the effects of a thermoneutral environment on heart rate variability, where mice exhibit a high sympathetic drive to maintain a normal core temperature (37°C) under standard laboratory conditions (20°C) (15, 36).” Please provide more details about how your model implemented the factor of temperature, and why and how specifically “This modeling choice aligns with earlier experimental findings”

RE: In our original manuscript, SNA was modeled with no circadian variations, constrained by experimental measurements by Barazi et al. 2021. This modeling choice was supported by previous experimental studies reporting a high sympathetic drive, potentially leaving less room for its additional time-of-day variations, to maintain a normal core temperature (~37°C) under laboratory conditions at 20°C. We have provided more details as follows:

Page 25, methods: “*Furthermore, SNA (Fig 1; yellow dot) was implemented to mimic isoproterenol (ISO) effects by modifying the kinetics and/or conductance of Ist, INa1.1, ICaT, ICaL, IK1, IKs, IKr, Ito, and the activities of JRel and Jup, using parameter settings as previously described (see Supporting Information Table S1 for more details) (48). The administration of ISO was scaled to align with experimental measurements of HR acceleration under PNA blockade conditions (BT=37°C) (Fig 2A; yellow dots) (38). A combination of LCR and ISO administration is enough to generate a circadian amplitude (~3%) in SANC FR as measured in the experiments (38), allowing little room for additional in-phase circadian variations in SNA. Therefore, SNA in this study was modeled with no circadian variations when CRBT is not present (BT=37°C). This modeling choice aligns with earlier experimental findings indicating a high sympathetic drive in mice, potentially limiting additional time-of-day variations to maintain a normal core temperature (37°C) under standard laboratory conditions (20°C) (26, 74).”*

In addition to this modeling choice regarding the factor of temperature, we’ve also extended our model to incorporate the circadian rhythm of body temperature (CRBT) in both adult and aged mice, as suggested by Reviewer 3, with full model development, validation, and simulation protocols provided in our revised methods section (see our response to reviewer 3, comment 3).

5. “We modeled LCR as a diurnal variation in the expression level of I_HCN_, but it might not be the only mechanism underlying LCR in SANC.” Yes, sure but this is a major study limitation of the study and should be discussed in more detail. You perform parametric sensitivity analysis for both CO and MO (i.e. Ca and membrane oscillators), but model LCRs only as one component in MO.

RE: Indeed, to model LCR in SANC with only I_HCN_ is a limitation in our study. Day-night rhythms have been reported in the transcription level of several other K^+^ channels; however, to our knowledge, I_HCN_ is most characterized in terms of circadian variations in its expression level and functional current. Thus, we used circadian rhythms in I_HCN_ to represent LCR in SANC. In addition, it has been also shown that CaMKII may also exhibit circadian variations. However, the baseline model used in our study lacks a fully detailed description of CaMKII signaling, and developing such signaling regulatory effects is beyond the scope of our current study. Nonetheless, exploring the influence of additional K+ currents and CaMKII could be a future direction for future model development.

Page 23, discussion: “*We have made several assumptions for simplicity in developing our model that may impact the generalizability and interpretation of our model simulations.* Our adult model was calibrated to experimental data in anesthetized mice (38) assuming limited circadian activities in SNA (26), which may potentially underestimate the role of SNA in SANC flexibility in free-moving mice living in a thermoneutral environment (74). Our aged model was parameterized based on a cross-sectional experimental dataset in >32 months old mice, thus lacking the capacity to capture functional trajectories throughout the aging process. *We modeled LCR as a diurnal variation in the expression level of IHCN, but it might not be the only mechanism underlying LCR in SANC. For example, a day/night difference has been reported in several K+ channels and in Ca2+/calmodulin-dependent protein kinase II delta expression (38).* We modeled CRBT as a simplified sinus function that peaks at ZT18, while actual experimental recordings of CRBT could feature additional complexities with surges in BT immediately after waking up and before sleep, and potential phase advances in aged mice (17). Inherited from the baseline model (48, 51, 75), specific molecular identities of several ion channels (e.g., Ist, INab, ICab) remain unknown, and a biochemical description of beta-adrenergic and cholinergic signaling pathways and their interactions is absent. In addition, our model is limited by the lack of a detailed transcription-translation feedback loop model for LCR in SANC, owing to the scope of this study and the limited availability of experimental data. Additional model development or experimental studies would be helpful to further advance our understanding of circadian SANC function and dysfunction, e.g., non-additive interactions between SNA and PNA(6), the mechanistic coupling between the ANS and LCR (76), trajectory of functional aging (77), and entrainment dynamics between the master circadian clock and LCR during jet lag or with shift work disorder (78).”

6. How were CO-MO parameter space maps created in Figs 3 and 4? You showed MO changes from 0 to 1 and CO also from 0 to 1. Which specific model parameters were varied, in what specific range, and why were these parameters and ranges chosen?

RE: In Fig 3 and Fig 4, each CO-MO parameter space map was generated by reducing the maximal rate of SERCA (Pup) (a CO parameter) and the maximal conductance of ICaL and ICaT (MO parameters) from their original values to zero, using a step size of 1%, resulting in a total of 100 × 100 simulations. The original values for these CO and MO parameters were defined as previously described (39), with Pup= 0.04 mM/ms, GCaL= 0.018 nS/pF and GCaT = 0.013956 nS/pF. The steady-state FR in BPM was calculated for each simulation and color-coded to create the map. We chose to use Pup to represent CO in alignment with previous modeling studies (Maltsev and Lakatta 2009, Kurata et al. 2012), regarding parameters to represent MO, both I_CaL_ and I_NCX_ has been employed in earlier studies (Maltsev and Lakatta 2009, Kurata et al. 2012). Given the importance of I_CaT_ in mouse SANC (Mesirca et al 2014), we selected both I_CaL_ and I_CaT_ to represent MO. This choice aims to reduce the dependency of our simulation results on the properties and mathematical formalism of a specific ion channel.

Page 13, results: “To achieve this, we simulated CO-MO parameter space maps at ZT6 and ZT18 under control conditions in adult model (Fig 3A). *Specifically, each CO-MO parameter space map was generated by reducing the maximal rate of SERCA (Pup) (a CO parameter; along the vertical axis) and the maximal conductance of ICaL and ICaT (MO parameters; along the horizontal axis) from their original values to zero using a step size of 1%, resulting in a total of 100 × 100 simulations.* The original values for these CO and MO parameters were defined with Pup= 0.04 mM/ms, GCaL= 0.018 nS/pF and GCaT = 0.013956 nS/pF as previously described (48). The steady-state FR in BPM was computed for each simulation and color-coded to create the map.”

Page 29, methods: “*A 12h:12h light/dark lighting regime was implemented as in the experimental studies (19, 38). FR (Fig 2-5) was calculated as the averaged FR of the last 10 seconds of simulation after reaching steady-state, with model implementation and initial conditions previously described by Ding et al. (48). Each CO-MO parameter space map (Fig 3-4) (37) was reproduced by reducing Pup (CO) and the maximal conductance of ICaL and ICaT (MO) from their original values (48) to zero, using a step size of 1%, resulting in a total of 100 × 100 simulations. Regarding the choice of parameters to represent CO and MO, respectively, we chose Pup to represent CO as often used in previous modeling studies (37, 54, 82). Both ICaL and INCX have been used to represent MO in earlier studies (37, 54, 82). Given the importance of ICaT in mouse SANC (51, 83), we chose to use ICaL and ICaT to represent MO to ensure that our simulation results are less dependent on the properties and mathematical formalism of a given single ion channel. Each simulation was color-coded by steady-state FR in BPM. For each CO-MO map, the border between no-firing and irregular firing regions (Fig 3-4; yellow lines) was identified by segregating zero and non-zero values of FR. Additionally, the border between irregular and rhythmic firing regions (Fig 3-4; blue lines) was established by pinpointing the location of the first local trough along the direction of CO and MO reduction.”*

7. Explain pattern disruption (black band in the middle) in Fig.3 for ZT18 in SNAB and ANSB. There are also clear disruptions of BPM within rhythmic firing areas between the black area and blue line. This may indicate the way you changed model parameters in the sensitivity analysis for the MO and CO between 0 and 1, or possibly may indicate problems with your model integration or automatic analysis of the data. Please check and explain.

RE: Many thanks for your comments and suggestions. We’ve checked our model integration and analysis, and the pattern disruption in our original manuscript was indeed a model analysis error. We have now fixed this issue, extended our models according to other comments and suggestions, reproduced Fig 3, and revised our manuscript accordingly.

Page 15, results:

**Fig 3. The *alliance of ANS, CRBT and LCR* is essential to *achieve circadian flexibility and performance while preserving robustness* in SANC automaticity.** (A) CO-MO parameter space maps color-coded by FR in BPM at ZT6 and ZT18 under control conditions in adult model. *(B) Steady-state SANC membrane potential oscillations using parameter settings sampled from panel (A; labeled 1 to 6). (C-G) CO-MO parameter space maps at ZT6 and ZT18 under (CRBT-/-) (C), (CRBT-/-; LCR-/-) (D), (CRBT-/-; ANS-/-) (E), (CRBT-/-; SNA-/-) (F), and (CRBT-/-; PNA-/-) (G) conditions. (H-J) Quantification of SANC flexibility (H), performance (I), and robustness (J) under various conditions. (K) Normalized changes in SANC flexibility, performance, and robustness compared to the control conditions in adult model.*

Page 14, results: “……*Similarly, without CRBT and ANS, SANC flexibility (19BPM) and performance (416BPM) are substantially impaired, with a minor reduction in averaged SANC robustness (52%). Additionally, without CRBT and SNA, SANC performance (382BPM) and robustness (averaged at 33%) are preferentially impaired over flexibility (50BPM). On the other hand, without CRBT and PNA, both performance (515BPM) and robustness (averaged at 63.5%) are markedly enhanced, while SANC flexibility (30BPM) is further reduced. These findings suggest that SNA preferentially enhances performance and robustness, while PNA inclines to amplify flexibility at the expense of both performance and robustness (Fig 3K). As a result, ANS promotes both SANC flexibility and performance, without compromising robustness. In addition, CRBT and LCR may act as primary and secondary boosters for SANC flexibility and performance (Fig 3K).”*

8. What algorithm was used to analyze the simulation data in Figs 3 and 4? In other terms, how did you get BPM in each simulation trace? This may make a big difference in the results, depending how you define AP, i.e. how you separate AP from a Vm oscillation near 0 mV.

RE: We used the MATLAB implementation and algorithm of the baseline model (Ding et al. 2022) to calculate BPM for each simulation trace; more specifically, firing rates were determined by averaging firing rates over the last 10 seconds of simulation once steady-state was achieved. The algorithm employed by Ding et al. utilizes a threshold function to distinguish a full AP from a Vm oscillation near 0 mV, and it's sufficient for our study to identify regions with different firing patterns in CO-MO parameter space maps.

Page 29, methods: “…… *FR (Fig 2-5) was calculated as the averaged FR of the last 10 seconds of simulation after reaching steady-state, with model implementation and initial conditions previously described by Ding et al. (48)……*

9. In Figs 3 and 4 the black area indicates: “Chaotic or no firing”. There is a big difference between chaotic vs. no firing. Please separate “Chaotic” and “no firing”. Also, in many panels (e.g., Fig 3 ZT18-control) there is a mosaic pattern of BPM between the blue line and black area. Is this a mosaic pattern chaotic firing? If so, why then it is not within the black area that includes chaotic firing as per your description? It’s important to provide clear definitions for chaotic and rhythmic firing in your analysis.

RE: Indeed, we agree with the reviewer that the labeling in our original Figs 3 and 4 could be misleading, and there is a big difference between chaotic vs no firing. To clarify this, we have extensively revised our Fig 3 (see our response to comment 7) and Fig 4, to separate no firing and irregular firing regions with an additional border (yellow). In addition, we also included a new Fig 3B (see our response to comment 7) to illustrate representative membrane oscillations with different parameter settings.

Page 13, results: “In the CO-MO parameter space maps, *borders between no firing, irregular firing, and rhythmic firing regions were denoted by yellow and blue lines, respectively (Fig 3A)..……”*

Page 17, results:

**Fig 4**. **Quantitative dissection of SANC pacemaking dysfunction in aging.** *(A-E) CO-MO parameter space maps at ZT6 and ZT18 under aged (A), aged MO (B), aged PNA (C), aged CO (D), and aged MO+PNA+CO (E) conditions. (F-H) Quantitative differences in flexibility (F), performance (G), and robustness (H) under various aging conditions. (I) Normalized changes in SANC flexibility, performance, and robustness compared to the control conditions in adult model.*

10. What was your rational to perform sensitivity analysis with the key model parameter reduced exactly by 25% in Fig. 5B?

RE: For sensitivity analysis in our original manuscript, a 25% reduction was arbitrarily chosen for perturbing individual parameters in Fig 5B. In the revised manuscript, we have extensively extended our adult and aged models to incorporate CRBT, aging-dependent changes in Ca signaling, ion channel remodeling with more comprehensive targets, and CRBT and PNA disruption in aged mice. To avoid arbitrary choices and align the sensitivity analysis with our extended models in adult and aged mice, we have redesigned our new Fig 5. This figure now quantitatively compares the relative contributions of each model component with full inhibition (no requirement for reduction percentages) in determining SANC flexibility and performance in both adult and aged mice.

Page 18, results: “*To dissect key mechanisms underlying circadian patterns in SANC, we assessed the relative contributions of each module in the model to circadian flexibility and performance of SANC. Specifically, we quantified how much the flexibility or performance of SANC was perturbed after fully inhibiting ANS, CRBT, or LCR in the adult model (Fig 5A) and aged model (Fig 5B). Furthermore, as the role of intracellular Na+ ([Na]i+) accumulation in regulating cardiac excitation patterns has been previously reported (49-51), we also clamped the time-of-day [Na]i+ content at its steady-state concentration at ZT6 to evaluate its potential role in regulating the flexibility and performance of SANC.*

*In adult model (Fig 5A), while ANS inhibition significantly reduces the circadian flexibility (-78%) and performance (-20.2%) of SANC, the effects of CRBT or LCR inhibition are secondary in reducing circadian flexibility (-46.5% and -6.7%, respectively) and performance (-19.2% and -2.8%, respectively). Furthermore, [Na]i+ clamping exerts an enhancing effect on both SANC flexibility (-10.6%) and performance (-2.9%), attributed to a higher [Na]i+ content at faster FR. However, in aged model, different mechanisms underlying the circadian flexibility and performance of SANC were observed (Fig 5B). While the effects of ANS inhibition remain most sensitive, leading to a reduction in SANC flexibility (-77.9%) and performance (-15.9%), the effects of CRBT and LCR inhibition are enhanced, causing a reduction in flexibility (-56.6% and -31.9%, respectively) compared to the adult model (Fig 5A-5B). This enhanced effect may be attributable to aging-dependent PNA impairment. In addition, the effect of [Na]i+ clamping becomes diminished and reversed in the aged model (Fig 5B) compared to the adult model (Fig 5A).*

*To further quantify mechanisms underlying aging-dependent reduction in SANC flexibility and performance in aged model, we additionally quantified the extent to which either flexibility or performance of SANC was perturbed by fully inhibiting MO remodeling, PNA impairment, CO alteration and CRBT disruption using the aged model (Fig 5C). The reduction of SANC flexibility in aging is mostly dampened by the inhibition of PNA impairment (-30.8%) and MO remodeling (-26.4%), with a secondary contribution of the inhibition of CRBT disruption (-18.3%), and a negligible effect by the inhibition of CO alteration (-1%). However, the reduction of SANC performance in aging is predominantly weakened by the inhibition of MO remodeling (-118.4%), with secondary contributions of the inhibition of CO alteration (-14.2%) and CRBT disruption (-8.4%). On the other hand, the reduction of SANC performance in aging is promoted by the inhibition of PNA impairment (9.6%).”*

Page 19, results:

**Fig 5**. **Fig 5. Distinct mechanisms underlying circadian patterns of SANC FR in adult and aged mice.** *(A-B) Relative contributions to SANC flexibility and performance are quantified by selective inhibition of each module in the adult (A) and aged (B) models. (C) Relative contributions to aging-dependent reduction in SANC flexibility and performance are quantified by selective inhibition of each aging-dependent change in the aged model (red box).*

11. How did you define the specific model parameter changes in aging in Fig 5A? Was your choice based on experimental studies? If so, please cite the respective literature in the figure legend. If not, then explain your reasoning for the parameters you use in the simulations.

RE: Apologies for any confusion. Our aging model was developed based on the most relevant experimental studies. To clarify this issue and fully explain our modeling choices regarding aging, we have revised Fig 2 and provided an extensive description in our revised methods section.

Page 27, methods: *“Aging-dependent ion channel remodeling (Fig 1; red dot) was calibrated to be consistent with electrophysiological measurements by Larson et al. (25), Tellez et al. (39) and Liu et al. (40) as follows:*

$I_{CaT,aging}=a_{cat}\times I_{CaT}$*,*

$I_{CaL,aging}=a_{cal}\times I_{CaL}$*,*

$I_{HCN,aging}=a_{hcn}\times I_{HCN}$*,*

$I_{NaK,aging}=a_{nak}\times I_{NaK}$*,*

$I_{NCX,aging}=a_{ncx}\times I_{NCX}$*,*

$I_{Kr,aging}=a_{hcn}\times I_{Kr}$*,*

$$I_{Ks,aging}=a_{hcn}\times I_{Ks}$$

*where a_cat_, a_cal_, a_hcn_, a_nak_, a_ncx_, a_kr_, a_ks_ are the scaling factors for I_CaT_, I_CaL_, I_HCN_, I_NaK_, I_NCX_, I_Kr_ , and I_Ks_ under aging conditions, respectively. Moreover, the aging-dependent shift of the activation midpoint of I_HCN_ was modeled as the following:*

$V_{HCN, aging}=V_{HCN}+v_{hcn}$*,*

*where V_HCN_ is the activation midpoint of I_HCN_ in adult model, and v_hcn_ is the activation shift of I_HCN_ in aged model.*

*Aging-dependent Ca^2+^ cycling dysfunction (Fig 1; red dot) in SERCA (J_up_) and Ryanodine receptor Ca release (J_Rel_) were calibrated according to previous experimental measurements by Liu et al. (40) as follows:*

$$J_{up,aging}=a_{up}\times J_{up}$$

$$J_{Rel,aging}=a_{rel}\times J_{Rel}$$

*where a_up_, a_rel_ are the scaling factors for J_up_, J_Rel_ under aging conditions, respectively. Moreover, the aging-dependent increase in the ratio between phospholamban and SERCA (40) was modeled by an increase to the original value of cytoplasmic Ca^2+^ sensitivity coefficient (K_mf_) in the baseline model (48) as follows:*

$$K_{mf,aging}=a_{kmf}\times K_{mf}$$

*where a_kmf_ is the scaling factor for K_mf_ under aging conditions.*

*In addition, aging-related PNA impairment (Fig 1; red dot) was introduced by a major reduction in PNA to be consistent with previous experimental studies (41) as follows:*

$${CCh}_{aging}=a_{cch}\times CCh$$

*where a_cch_ is the scaling factor of CCh under aging conditions. SNA was assumed to be unchanged in aged mice as reported by Larson et al. (25).*

*Moreover, aging-dependent CRBT disruption was modeled by a reduction in both averaged time-of-day BT and circadian amplitude of BT in aged mice as previously reported (17). Specifically:*

${BT}_{aging}={BT}_{a,aging}+A_{BT,aging}\times\cos(\frac{2\pi\times(T+6)}{T_{BT}})$*,*

*where BT_a,aging_ is the reference BT in aged model, and A_BT,aging_ is the circadian amplitude of BT in aged model.”*

Page 12, results:

**Fig 2**. **Quantitative reconstruction of diverse circadian patterns in SANC FR under various conditions.** …… *(H-I) In aged model, simulated maximal FR reduction (25%; red dots) (H) is consistent with experimental findings (I) (25)”*

Page 11, results: “*Next, expanding on our model with ANS and LCR (Fig 2B; grey line), we further incorporated CRBT (Fig 1) as a circadian function to reproduce the circadian patterns of BT in adult mice, with an averaged time-of-day BT of 37°C, and a circadian amplitude of 1°C. Then, we incorporated temperature-dependent factors to adjust gating kinetics and conductance of ion channels as previously described (10, 45). For ionic pumps, exchangers and intracellular ionic diffusion parameters, their temperature-dependent behaviors were simulated by scaling their maximal capacity or original value with Q10. Furthermore, we modeled the temperature-dependent activities of SNA and PNA by introducing linear adjustments based on experimental measurements (14) (see Methods for details). With CRBT, simulated circadian amplitude of SANC FR was further enhanced (Fig 2F; grey dots), yielding a Q10 value of 2 as previously reported in experimental studies (Fig 2G) (14, 15). We refer this model with CRBT, ANS, and LCR as adult model throughout this work.”*

12. I found it very difficult to read the paper. It seems like cryptography with numerous abbreviations: FR, MO, CO, SNA, ANS, LCR, SANC, AP, NUC, PNA, PNA, SNAB, ANSB, HR, iHR, DCN, GPCR, PKA, AC, SR, HCN, SERCA. P-S, S-P, BPM. Further, many abbreviations are not explicitly given like ZT6 and ZT18. Please reduce abbreviations to a minimum to make the text readable. For example, why not write simply firing rate instead of FR or Ca oscillator instead of CO, etc.? Also please include a table defining all terms.

RE: Many thanks for your comments and suggestions regarding terms and abbreviations used in our original manuscript. To address this issue, we have reduced the number of abbreviations used in our revision, including ECG, GPCR, AC, SNAB, PNAB, ANSB, LCB, iHR, NUC, CYT, SS, CaMKII, TTFL etc., to keep these abbreviations to a minimum as suggested.

Page 4, introduction: “The mammalian heart exhibits robust circadian rhythms in various cardiac function indices, such as heart rate (HR) and *electrocardiogram* waveforms (1).”

Page 5, introduction: “……However, under *ANS blockade* conditions, further studies demonstrate the significant contribution of LCR in promoting diurnal variations in the *intrinsic* HR (*HR with no autonomic control*) (22, 23).

Page 23, discussion: “…… For example, a day/night difference has been reported in several K+ channels and in *Ca2+/calmodulin-dependent protein kinase II delta* expression (38). We modeled CRBT as a simplified sinus function that peaks at ZT18, while actual experimental recordings of CRBT could feature additional complexities with surges in BT immediately after waking up and before sleep, and potential phase advances in aged mice (17). Inherited from the baseline model (48, 51, 75), specific molecular identities of several ion channels (e.g., Ist, INab, ICab) remain unknown, and a biochemical description of beta-adrenergic and cholinergic signaling pathways and their interactions is absent. In addition, our model is limited by the lack of a detailed *transcription-translation feedback loop* model for LCR in SANC, owing to the scope of this study and the limited availability of experimental data……”

RE: Regarding CO and FR, given their frequent usage (>20, and >50 times respectively) throughout the manuscript, we have opted to retain them and enhance their clarity in Fig 1 and Fig 2. Specifically, we listed key term definitions, such as CO and MO in Fig 1; we spelled out heart/firing rates in Fig 2 instead of using HR/FR as suggested by the reviewer. Additionally, we have provided Table 2 to explicitly define frequently used abbreviations. Conversely, for less used abbreviations, encompassing subcellular compartments, membrane ionic channels, exchangers, pumps, and intracellular fluxes, we have directly provided their definitions in the figure or figure legend to enhance reader comprehension (Fig 1).

Page 7, introduction:

**Fig 1**. **Model schematic for ANS, CRBT and LCR in mouse SANC ……** *INa1.1, Na+ channel isoform Nav1.1 current; INa1.5, Na+ channel isoform Nav1.5 current; Ist, sustained inward Na+ current; INab, background Na+ current; Ito, transient component of 4-Aminopyridine-sensitive current; Isus, sustained component of 4-Aminopyridine-sensitive current; IKr, rapid delayed rectifying K+ current; IKs, slow delayed rectifying K+ current; IK1, inward rectifier K+ current; IKb, background K+ current; IKAch, muscarinic K+ current; ICaL, L-type Ca2+ channel current; ICaT, T-type Ca2+ channel current; INCX, Na+/Ca2+ exchanger current; ICab, background Ca2+ current; INaK, Na+/K+ pump current; IHCN, hyperpolarization-activated cyclic nucleotide–gated channel current; JSR, junctional sarcoplasmic reticulum; NSR, network sarcoplasmic reticulum; Jdiff, Ca2+ diffusion flux from subspace to cytoplasm compartment; JRel, Ca2+ release from JSR to subspace compartment; Jtr, Ca2+ transfer flux from NSR to JSR; Jup, SERCA Ca2+ pump flux*.

Page 12, results:

**Fig 2**. **Quantitative reconstruction of diverse circadian patterns in SANC FR under various conditions**…….

Page 8, introduction:

*Table 2 Definitions of Non-standard Abbreviations*

| **Abbreviations** | **Definitions** |
| --- | --- |
| ANS | Autonomic nervous system |
| AP | Action potential |
| BPM | Beats per minute |
| BT | Body temperature |
| cAMP | Cyclic adenosine monophosphate |
| CCh | Carbachol |
| CO | Ca^2+^ oscillator |
| CRBT | Circadian rhythm of BT |
| FR | Firing rates of a single sinoatrial nodal cell |
| HR | Heart rates |
| G_x_ | Maximal conductance of ion channel x |
| ISO | Isoproterenol |
| LCR | Local circadian rhythmicity |
| MO | Membrane oscillator |
| PNA | Parasympathetic nervous activities |
| P_up_ | Maximal rate of SERCA Ca^2+^ pump |
| PKA | Protein kinase A |
| SANC | Sinoatrial nodal cells |
| SCN | Suprachiasmatic nucleus |
| SERCA | SR Ca^2+^-ATPase |
| SNA | Sympathetic nervous activities |
| SR | Sarcoplasmic reticulum |
| ZT | Zeitgeber time |

13. Ist has a strong contribution to your model, whereas molecular identity of this current remains unknown. The same is about INab, and Cab. Please explain.

RE: We agree with the reviewer that molecular identities of ionic currents including I_st_, I_Nab_ and I_Cab_ remain unknown, and they are inherited from the baseline model developed by Kharche et al. 2011 and Morotti et al. 2021. While these currents may require updates if experimental data are available, this is beyond the scope of the present study. In our revised manuscript, our primary focus has been on dissecting the roles of different model components (such as ANS, CRBT or LCR) rather than individual ionic currents in delineating circadian patterns of SANC firing rates. To address this issue, we have revised our discussion section accordingly.

Page 23, discussion: “We have made several assumptions for simplicity in developing our model that may impact the generalizability and interpretation of our model simulations. …… *Inherited from the baseline model (48, 51, 75), specific molecular identities of several ion channels (e.g., Ist, INab, ICab) remain unknown, and a biochemical description of beta-adrenergic and cholinergic signaling pathways and their interactions is absent. In addition, our model is limited by the lack of a detailed transcription-translation feedback loop model for LCR in SANC, owing to the scope of this study and the limited availability of experimental data.* Additional model development or experimental studies would be helpful to further advance our understanding of circadian SANC function and dysfunction, e.g., non-additive interactions between SNA and PNA(6), the mechanistic coupling between the ANS and LCR (76), trajectory of functional aging (77), and entrainment dynamics between the master circadian clock and LCR during jet lag or with shift work disorder (78).”

14. In Fig.1 only 3 currents are affected by aging (ICaL, ICaT, I HCN), all other 13 current remain unchanged, including currents of major functional importance, such as NCX, NaK, KACh, Ito, Na currents. Please explain why only three currents change with aging in your model. What is known about how the other 13 currents change with aging. If some or all these currents do change with aging, your results would likely be different?

RE: Many thanks for your comments and suggestions regarding ionic currents affected by aging. To address this issue, we’ve extended our aging model to account for more comprehensive aging-dependent ion channel remodeling (I_CaL_, I_CaT_, I_HCN_ , I_NaK_, I_Kr_, I_Ks_ and I_NCX_ (Larson et al. 2013, Tellez et al. 2011, Liu et al. 2014)), and revised our methods section accordingly.

Page 27, methods: *“Aging-dependent ion channel remodeling (Fig 1; red dot) was calibrated to be consistent with electrophysiological measurements by Larson et al. (25), Tellez et al. (39) and Liu et al. (40) as follows:*

$I_{CaT,aging}=a_{cat}\times I_{CaT}$*,*

$I_{CaL,aging}=a_{cal}\times I_{CaL}$*,*

$I_{HCN,aging}=a_{hcn}\times I_{HCN}$*,*

$I_{NaK,aging}=a_{nak}\times I_{NaK}$*,*

$I_{NCX,aging}=a_{ncx}\times I_{NCX}$*,*

$I_{Kr,aging}=a_{hcn}\times I_{Kr}$*,*

$$I_{Ks,aging}=a_{hcn}\times I_{Ks}$$

*where a_cat_, a_cal_, a_hcn_, a_nak_, a_ncx_, a_kr_, a_ks_ are the scaling factors for I_CaT_, I_CaL_, I_HCN_, I_NaK_, I_NCX_, I_Kr_ , and I_Ks_ under aging conditions, respectively. Moreover, the aging-dependent shift of the activation midpoint of I_HCN_ was modeled as the following:*

$V_{HCN, aging}=V_{HCN}+v_{hcn}$*,*

*where V_HCN_ is the activation midpoint of I_HCN_ in adult model, and v_hcn_ is the activation shift of I_HCN_ in aged model.*”

RE: With this extended description of aging-dependent ion channel remodeling, we’ve updated our model schematic (Fig 1; with additional aging targets of ion channels, exchangers, and pumps), simulation results, and the corresponding portion of the manuscript accordingly.

Page 7, introduction:

**Fig 1**. **Model schematic for ANS, CRBT and LCR in mouse SANC.**...... *In aged mice, SANC pacemaking function is disrupted by aging-dependent ion channel remodeling, intracellular Ca2+ cycling alternation, PNA impairment, and CRBT disruption (red dots) (25, 26, 39-41)..*.....*”*

15. In Fig.1 Jup and Jrel (and the entire Ca oscillator module - CO) are not affected by aging. This is not correct. There is evidence that Ca signaling (SERCA, RyR, NCX) deteriorates with age, see e.g. Liu et al. Am J Physiol Heart Circ Physiol. 2014;306(10):H1385-97. Thus, your model does not reproduce a major change in the Ca oscillator. Please explain or make respective additional simulations.

RE: Many thanks for your comments and suggestions regarding Ca signaling dysfunction in aging. To address this issue, we’ve now extended our aging model to account for aging-dependent changes in NCX, RyR Ca release, and SERCA as reported by Liu et al. 2014, and revised our methods section accordingly (see our response to comment 14).

Page 28, methods: “*Aging-dependent Ca^2+^ cycling dysfunction (Fig 1; red dot) in SERCA (J_up_) and Ryanodine receptor Ca release (J_Rel_) were calibrated according to previous experimental measurements by Liu et al. (40) as follows:*

$$J_{up,aging}=a_{up}\times J_{up}$$

$$J_{Rel,aging}=a_{rel}\times J_{Rel}$$

*where a_up_, a_rel_ are the scaling factors for J_up_, J_Rel_ under aging conditions, respectively. Moreover, the aging-dependent increase in the ratio between phospholamban and SERCA (40) was modeled by an increase to the original value of cytoplasmic Ca^2+^ sensitivity coefficient (K_mf_) in the baseline model (48) as follows:*

$$K_{mf,aging}=a_{kmf}\times K_{mf}$$

*where a_kmf_ is the scaling factor for K_mf_ under aging conditions.*”

RE: With this extended description of aging-dependent Ca signaling dysfunction, we’ve updated our model schematic (Fig 1; with additional aging targets in Ca cycling) (see our response to comment 14), simulation results, and the corresponding portion of the manuscript accordingly.

***Minor Comments***

1. Please clearly define Robustness, Flexibility, and Performance, and explain why those definitions are reasonable.

RE: Many thanks for your comments and suggestions. To address this issue and improve the clarity of our manuscript, we’ve included a new Table 1 to clearly define robustness, flexibility, and performance in the context of circadian rhythms in heart rates, and have revised our manuscript accordingly to further explain why those definitions are reasonable. In addition, we also revised our figures to label their technical definitions associated with each CO-MO parameter space map.

Page 6, introduction:

Table 1 Glossary of Circadian Rhythms in HR

| **Term** | **Explanation** |
| --- | --- |
| Circadian rhythms | Innate, roughly 24-hour biological cycles that regulate various physiological and behavioral processes in living organisms |
| Robustness | The heart’s capability to sustain rhythmic HR consistently throughout the diurnal cycle, irrespective of external factors or conditions |
| Performance | The heart’s capacity to achieve the maximum HR under conditions of increased demand or stress |
| Flexibility | The heart’s capacity to produce a diverse range of HR throughout the day |

Page 6, introduction: “Regulated jointly by the *ANS, CRBT, and LCR* (Fig 1), circadian rhythms in HR display intriguing properties of *robustness, performance, and flexibility (Table 1). Despite changes in external conditions, they remain robust, ensuring the generation of rhythmic HR patterns throughout the day. They are capable of generating maximum HR (performance) in response to heightened demand or stress, often occurring after waking up. Moreover, they demonstrate flexibility, accommodating a broad range of pacing frequencies throughout a day, so that time-of-day cardiac outputs can be optimized under diverse physiological conditions, such as sleep/awake or inactive/active states.* However, how *ANS, CRBT, and LCR* interact to facilitate circadian flexibility and performance in cardiac pacemaking function with robustness remains unclear. Furthermore, their vulnerability to adaptation under severe perturbations, such as aging (Fig 1; red dot), remains enigmatic (24-26).”

2. You wrote: “This AC cAMP-PKA signaling cascade leads to the phosphorylation of various targets, such as the L-type Ca2+channel (ICaL), Ryanodine receptor, and sarco/endoplasmic reticulum (SR) Ca2+ ATPase (SERCA)”. In fact, PKA activates SERCA by phosphorylation of phospholamban, is there any evidence that SERCA, itself becomes phosphorylated?

RE: Yes, we agree with you that PKA activates SERCA by the phosphorylation of phospholamban. To address this issue, we have revised our manuscript accordingly.

Page 4, introduction: “This cAMP-PKA signaling pathway leads to the phosphorylation of various MO targets, such as the L-type Ca2+ channel (ICaL), T-type Ca2+ channel (ICaT), and CO targets, including Ryanodine receptor, and *phospholamban that regulates the activity of SERCA* (Fig 1; yellow dot) (5).”

**Reviewer #2:**

The authors develop a mathematical model designed to predict the impact and importance of various factors (parasympathetic and sympathetic nervous system and intrinsic gene changes) in circadian regulation of heart rate (HR). The authors conclude that “our work shed light on their critical synergistic interactions in regulating time-of-day cardiac pacemaking function and dysfunction, which may help to identify potential therapeutic targets within the circadian clock for the prevention and treatment of cardiac arrhythmias.”

Strengths: The topic is of some general interest.  A strength of the paper is the attempt to reproduce and incorporate recent experiments data in anesthetized mice.

Weaknesses: Generally, the presentation of the model and its results could be improved.  Specific comments follow. 

 **Specific comments**

1.  The Abstract could be more informative.  Terms like robustness, performance and flexibility are not accessible to the general reader.  In fact, even a person with experience expertise in the field of cardiac physiology would find it challenging to understand the basis and meaning of the conclusions. For example, the authors state “Our model underscores the indispensable synergy among SNA, PNA, and LCR in preserving circadian flexibility, robustness, and performance in SANC”.  What specifically is flexibility etc?

RE: Many thanks for your comments regarding the abstract and terms used in our manuscript. We agree with the reviewer that terms like robustness, performance, and flexibility may not be readily accessible. To address this concern, we have revised our abstract and manuscript accordingly. In addition, we have included a new Table 1 to clearly define the glossary used in our study (see our response to reviewer 1, minor comment 1).

Page 2, abstract: “Strong circadian (~24h) rhythms in heart rate (HR) are critical for flexible regulation of cardiac pacemaking function throughout the day. While this circadian flexibility in HR is sustained in diverse conditions, it declines with age, accompanied by reduced maximal HR performance. *The intricate regulation of circadian HR involves the orchestration of the autonomic nervous system (ANS), circadian rhythms of body temperature (CRBT), and local circadian rhythmicity (LCR), which has not been fully understood*. Here, we developed a mathematical model describing ANS, CRBT, and LCR in sinoatrial nodal cells (SANC) that accurately captures distinct circadian patterns in adult and aged mice. *Our model underscores how the alliance among ANS, CRBT, and LCR achieves circadian flexibility to cover a wide range of firing rates in SANC, performance to achieve maximal firing rates, while preserving robustness to generate rhythmic firing patterns irrespective of external conditions*. Specifically, while ANS dominates in promoting SANC flexibility and performance, CRBT and LCR act as primary and secondary boosters, respectively, to further enhance SANC flexibility and performance. Disruption of this alliance with age results in impaired SANC flexibility and performance, but not robustness This unexpected outcome is primarily attributed to the age-related reduction in parasympathetic activities, which maintains SANC robustness while compromising flexibility. Our work sheds light on the critical alliance of ANS, CRBT, and LCR in regulating time-of-day cardiac pacemaking function and dysfunction, offering insights into novel therapeutic targets for the prevention and treatment of cardiac arrhythmias.”

Page 6, introduction: “Regulated jointly by the *ANS, CRBT, and LCR* (Fig 1), circadian rhythms in HR display intriguing properties of *robustness, performance, and flexibility (Table 1). Despite changes in external conditions, they remain robust, ensuring the generation of rhythmic HR patterns throughout the day. They are capable of generating maximum HR (performance) in response to heightened demand or stress, often occurring after waking up. Moreover, they demonstrate flexibility, accommodating a broad range of pacing frequencies throughout a day, so that time-of-day cardiac outputs can be optimized under diverse physiological conditions, such as sleep/awake or inactive/active states.* However, how *ANS, CRBT, and LCR* interact to facilitate circadian flexibility and performance in cardiac pacemaking function with robustness remains unclear. Furthermore, their vulnerability to adaptation under severe perturbations, such as aging (Fig 1; red dot), remains enigmatic (24-26).*”*

2.  The details of the model need to better documented.  What method was used to solve the model shown in Figure 1?  What were the initial conditions? It would be very useful to show sample “oscillations”.  This could be used by the authors to illustrate/define what is meant by robustness, as well as possibly flexibility.

 RE: We used the ode15s solver in MATLAB to solve the model shown in Fig 1, and we used the same initial conditions as described in Ding et al., Elife, 2022. Many thanks for your suggestions to show sample oscillations to improve the readability of our manuscript and help illustrate the definitions of robustness, flexibility, and performance. To address this issue, we’ve extended our methods section to provide full details of model implementation, and revised our Fig 3 and Fig 4 to show sample “oscillations” (Fig 3B) with detailed labeling of robustness, flexibility, and performance for each CO-MO parameter space map (Fig 3A).

Page 30, methods: “*All model simulations were performed with parallel computing on a ThinkStation P620 tower workstation with an AMD Threadripper processor. Model codes were implemented and solved in MATLAB (Version: 9.13.0 (R2022b)) using ode15s, and will be provided for public download from https://github.com/Mathbiomed/CircSANC.*”

Page 15, results:

**Fig 3**. **The alliance of *ANS, CRBT and LCR* is essential to achieve circadian flexibility and performance *while preserving robustness* in SANC automaticity.** (A) CO-MO parameter space maps color-coded by FR in BPM at ZT6 and ZT18 under control conditions in adult model. *(B) Steady-state SANC membrane potential oscillations using parameter settings sampled from panel (A; labeled 1 to 6). (C-G) CO-MO parameter space maps at ZT6 and ZT18 under (CRBT-/-) (C), (CRBT-/-; LCR-/-) (D), (CRBT-/-; ANS-/-) (E), (CRBT-/-; SNA-/-) (F), and (CRBT-/-; PNA-/-) (G) conditions. (H-J) Quantification of SANC flexibility (H), performance (I), and robustness (J) under various conditions. (K) Normalized changes in SANC flexibility, performance, and robustness compared to the control conditions in adult model.*

3.  Was experimental data used to constrain the parameters in the model?  If not, how were various parameters like those that generated the LCR or the amplitude of the SNA and PNA?

 RE: Yes, we used the most relevant experimental data to constrain our adult and aged models. To address this issue, we’ve extensively extended our methods section to provide full details regarding model parameterization.

Page 24, methods: *“Autonomic, electrophysiologic, and circadian properties of cardiac function are species-dependent (26, 79). Given the availability of experimental data in mice, our model is constructed to be mouse-specific. We used the mathematical model of mouse SANC AP developed by Ding et al. (48, 51), based on the original work of Kharche et al. (75), as our baseline model. All baseline model definitions, equations, and parameter settings were unchanged from those previously described and implemented by Ding et al. (48). Due to the absence of circadian regulation in the baseline model, we extended it to quantitatively describe circadian variations in ANS, CRBT, and LCR for both adult and aged mice. Detailed descriptions of model equations are provided in the following section, along with all parameter values and settings listed in Table S1 (Supporting Information).*

*Incorporation of LCR into the baseline model*

*First, without considering CRBT, LCR was introduced and calibrated into the baseline model as a circadian variation in the I_HCN_ (Fig 1; green dot) to generate a weak circadian amplitude (~2%) in SANC FR as observed in adult mice under full ANS blockade with BT=37°C (Fig 2A; green dashed line) (19, 38). Specifically, to introduce circadian variations in I_HCN_ into the baseline model with ANS blockade, I_HCN_ was modified from its original form*$I_{HCN}=I_{HCN,Na}+ I_{HCN,K}$ *as follows:*

$I_{HCN}=\left( 1+ A_{LCR}\times cos (\frac{2\pi T}{T_{LCR}}) \right)\times\left( I_{HCN,Na}+ I_{HCN,K} \right)$*,*

*where A_LCR_ is the amplitude of LCR, T_LCR_ is the period of LCR, T is the current ZT in hours,* $I_{HCN,Na} \text{and} I_{HCN,K}$ *are the Na^+^ and K^+^ specific HCN channel currents, respectively (48). It should be noted that, while experimental recordings of HR with ANS blockade (38) show troughs at ZT12, experimental measurements of I_HCN_ currents (19) show troughs at ZT6. We speculated that this might be attributable to a phase shift in LCR due to a lack of external cues from the ANS. For this, when the ANS is present, I_HCN_ was modeled as follows:*

$$I_{HCN}=\left( 1+ A_{\text{LCR}}\times cos (\frac{2\pi\times(T+6)}{T_{LCR}}) \right)\times\left( I_{HCN,Na}+ I_{HCN,K} \right).$$

*Incorporation of the circadian regulation of ANS into the baseline model*

*Then, circadian PNA (Fig 1; blue dot) was added and calibrated as a circadian function of carbachol (CCh) concentrations, to reproduce the circadian amplitude (~7%) effects based on experimental data under SNA blockade conditions with BT=37°C (Fig 2A; blue dashed line) (38). For this, CCh was modified from its original form* $CCh={CCh}_{basal}$ *(48) as follows:*

$CCh={CCh}_{basal}+A_{CCh}\times\cos(\frac{2\pi\times(T-6)}{T_{CCh}})$*,*

*where CCh_basal_ is the basal concentration of CCh, A_CCh_ is the circadian amplitude of CCh, T_CCh_ is the period of CCh variations, and T is the current time in ZT hours. This circadian variation in CCh leads to the circadian variation in the muscarinic K^+^ current (I_KACh_) because I_KACh_ is regulated by CCh in the original baseline model as follows (Fig 1; blue dot) (48):*

$I_{KACh}=G_{KAch}\times w\left( V,CCh,t \right)\times(V-E_{K})$*,*

*where G_KACh_ is the maximal conductance of I_KACh_, V is the membrane potential in mV, t is current time in ms, w(V,CCh,t) is the voltage- and CCh-dependent channel activation function, and E_K_ is the K^+^ equilibrium potential in mV.*

*Furthermore, SNA (Fig 1; yellow dot) was implemented to mimic isoproterenol (ISO) effects by modifying the kinetics and/or conductance of I_st_, I_Na1.1_, I_CaT_, I_CaL_, I_K1_, I_Ks_, I_Kr_, I_to_, and the activities of J_Rel_ and J_up_, using parameter settings as previously described (see Supporting Information Table S1 for more details) (48). The administration of ISO was scaled to align with experimental measurements of HR acceleration under PNA blockade conditions (BT=37°C) (Fig 2A; yellow dots) (38). A combination of LCR and ISO administration is enough to generate a circadian amplitude (~3%) in SANC FR as measured in the experiments (38), allowing little room for additional in-phase circadian variations in SNA. Therefore, SNA in this study was modeled with no circadian variations when CRBT is not present (BT=37°C). This modeling choice aligns with earlier experimental findings indicating a high sympathetic drive in mice, potentially limiting additional time-of-day variations to maintain a normal core temperature (37°C) under standard laboratory conditions (20°C) (26, 74).*

*When SNA and PNA were both present, bidirectional modulation effects between PNA and SNA (P-S and S-P) (Fig 2B) were formulated and calibrated to account for the non-additivity effects (6, 42, 80, 81) and to be consistent with experimental measurements (Fig 2A; grey dots) (38). The non-additivity effects with ISO and CCh were implicitly modeled (e.g., via cAMP-PKA dependent pathways or crosstalk between sympathetic and parasympathetic nervous systems) by introducing the ISO effects at a given concentration of CCh as follows:*

$E_{ISO}=e_{iso}\times\frac{k_{p\_s}^{h}}{k_{p\_s}^{h}+{CCh}^{h}}$*,*

*where e_iso_ is the stand-alone ISO effects as previously described (48), k_p_s_ is the P-S modulation constant, and h is the Hill coefficient. Furthermore, CCh effects with ISO were introduced as*

$E_{CCh}=e_{cch}\times k_{s\_p}$*,*

*where e_cch_ is the CCh effects alone as previously described (48), and k_s_p_ is the S-P modulation scaling factor (e.g., to account for the cross-talk between SNA and PNA (6, 42, 80, 81)).*

*Incorporation of CRBT into the baseline model*

*CRBT (Fig 1) was added and calibrated as a circadian function of BT to reproduce the circadian patterns of BT in adult mice (with averaged time-of-day BT of 37°C and a circadian amplitude of 1°C) (38) (Fig 2F; grey dots). For this, BT was modified from its original form* $BT={BT}_{a}$ *(48) as follows:*

$BT={BT}_{a}+A_{BT}\times\cos(\frac{2\pi\times(T+6)}{T_{BT}})$*,*

*where BT_a_ is the reference BT, A_BT_ is the circadian amplitude of BT, T_BT_ is the period of BT variations, and T is the current time in ZT hours.*

*To account for the effects of CRBT for all ion channels (excluding ionic pumps and exchangers) in our model, we introduced two temperature-dependent factors* $\varphi\left( BT \right)$ *and* $\eta\left( BT \right)$*, to scale gating kinetics and conductance of ion channels respectively, as previously described in (10, 45) as follows:*

$\varphi\left( BT \right)=Q_{10}^{\frac{BT-{BT}_{a}}{10}}$*,*

$\eta\left( BT \right)=1+B\left( BT-{BT}_{a} \right)$*,*

*where BT is the current body temperature, BT_a_ is the reference body temperature, B is a scaling coefficient. For example, the temperature dependence of ion channel x was modeled as follows:*

$$\tau_{x,BT}=\frac{\tau_{x}}{\varphi\left( BT \right)}$$

$G_{x,BT}=\eta\left( BT \right)G_{x}$*,*

*where G_x_ and τ_x_ are the original conductance and gating time constant of ion channel x as in the baseline model, G_x,BT_ and τ_x,BT_ are the conductance and gating time constant of ion channel x at a given BT.*

*For ionic pumps, exchangers, and intracellular ionic diffusion parameters, their temperature-dependent behaviors were modeled by scaling their maximal activity or original value (if a constant) with* $\varphi\left( BT \right)$*. For SNA and PNA, it has been reported in earlier experiments (14) that around normal BT (e.g., 37°C), as BT increases, there is a linear increase and decrease in SNA and PNA, respectively, to promote HR acceleration. Thus, the temperature-dependent behaviors of SNA and PNA were modeled by scaling their activities with* $\eta\left( BT \right)$ *based on experimental findings (14).*

*Modeling aging-dependent alternations*

*Aging-dependent ion channel remodeling (Fig 1; red dot) was calibrated to be consistent with electrophysiological measurements by Larson et al. (25), Tellez et al. (39) and Liu et al. (40) as follows:*

$I_{CaT,aging}=a_{cat}\times I_{CaT}$*,*

$I_{CaL,aging}=a_{cal}\times I_{CaL}$*,*

$I_{HCN,aging}=a_{hcn}\times I_{HCN}$*,*

$I_{NaK,aging}=a_{nak}\times I_{NaK}$*,*

$I_{NCX,aging}=a_{ncx}\times I_{NCX}$*,*

$I_{Kr,aging}=a_{hcn}\times I_{Kr}$*,*

$$I_{Ks,aging}=a_{hcn}\times I_{Ks}$$

*where a_cat_, a_cal_, a_hcn_, a_nak_, a_ncx_, a_kr_, a_ks_ are the scaling factors for I_CaT_, I_CaL_, I_HCN_, I_NaK_, I_NCX_, I_Kr_ , and I_Ks_ under aging conditions, respectively. Moreover, the aging-dependent shift of the activation midpoint of I_HCN_ was modeled as the following:*

$V_{HCN, aging}=V_{HCN}+v_{hcn}$*,*

*where V_HCN_ is the activation midpoint of I_HCN_ in adult model, and v_hcn_ is the activation shift of I_HCN_ in aged model.*

*Aging-dependent Ca^2+^ cycling dysfunction (Fig 1; red dot) in SERCA (J_up_) and Ryanodine receptor Ca release (J_Rel_) were calibrated according to previous experimental measurements by Liu et al. (40) as follows:*

$$J_{up,aging}=a_{up}\times J_{up}$$

$$J_{Rel,aging}=a_{rel}\times J_{Rel}$$

*where a_up_, a_rel_ are the scaling factors for J_up_, J_Rel_ under aging conditions, respectively. Moreover, the aging-dependent increase in the ratio between phospholamban and SERCA (40) was modeled by an increase to the original value of cytoplasmic Ca^2+^ sensitivity coefficient (K_mf_) in the baseline model (48) as follows:*

$$K_{mf,aging}=a_{kmf}\times K_{mf}$$

*where a_kmf_ is the scaling factor for K_mf_ under aging conditions.*

*In addition, aging-related PNA impairment (Fig 1; red dot) was introduced by a major reduction in PNA to be consistent with previous experimental studies (41) as follows:*

$${CCh}_{aging}=a_{cch}\times CCh$$

*where a_cch_ is the scaling factor of CCh under aging conditions. SNA was assumed to be unchanged in aged mice as reported by Larson et al. (25).*

*Moreover, aging-dependent CRBT disruption was modeled by a reduction in both averaged time-of-day BT and circadian amplitude of BT in aged mice as previously reported (17). Specifically:*

${BT}_{aging}={BT}_{a,aging}+A_{BT,aging}\times\cos(\frac{2\pi\times(T+6)}{T_{BT}})$*,*

*where BT_a,aging_ is the reference BT in aged model, and A_BT,aging_ is the circadian amplitude of BT in aged model.”*

4.  The authors state that their simulations match closely the experimental data but the comparisons are limited to the amplitude of the fluctuations.  Figure 2 shows clearly that the mean HRs do not match between simulations and data.  Why do these not match?  Does this reflect the absence of constraining parameters with the experimental data?

RE: In Barazi et al. 2021, heart rates were estimated from telemetry ECG recordings; however, our model simulation of firing rates was conducted using a single sinoatrial nodal (SAN) cell model. Given the complexities at the tissue level, e.g., intercellular coupling, electrophysiological heterogeneity, and SAN-atrial tissue coupling, we consider heart rate as an organ-level property emergent from the collective behaviors of many coupled SAN cells, and firing rate is considered a cellular property of single SAN cells. As reported by Larson et al., PNAS, 2013, heart rates could be up to ~30% higher than firing rates measured in single SAN cells. Thus, we opted to compare normalized experimental data of heart rates and model simulation of firing rates to reconstruct various circadian patterns presented in Fig 2. To address this clarification, we have revised our Fig 2 and its legend accordingly.

Page 12, results:

**Fig 2**. **Fig 2. Quantitative reconstruction of diverse circadian patterns in SANC FR under various conditions.** (A) Averaged circadian HR fluctuations in anesthetized mice (BT=37°C) with 12-h light cycles before (control; grey dashed line) and after SNA blockade (blue dashed line), PNA blockade (yellow dashed line), and ANS blockade (green dashed line) (38). *Circadian fluctuations were fitted using a sine function as described in (38) and normalized to HR at ZT6 with ANS blockade*. (B) *Simulated circadian FR patterns in a single mouse SANC (BT=37°C; normalized to FR at ZT6 with ANS blockade) closely recapitulate experimental findings* (38). (C-D) *Model simulations accurately reproduce minimal and maximal time-of-day FR (C), and normalized circadian amplitudes of FR (D)* under control, SNA blockade, PNA blockade, and ANS blockade conditions. The normalized amplitude values are obtained by dividing circadian FR amplitudes in beats per minute (BPM) by the mean time-of-day FR. (E) *Either additive or unidirectional modulation effects alone are insufficient to accurately reproduce time-of-day FRs under the control conditions*……

 5.  The concept of “bidirectional modulation” need to be defined and the main features discussed.  A figure illustrating this interaction with actual oscillations would be helpful.

RE: Many thanks for your comments regarding bidirectional modulation. To address this issue, we have revised our Fig 2to highlight the differences between additive, unidirectional, and non-additive modulations (Fig 2D) (see our response to comment 4). In addition, we also included a more detailed description regarding bidirectional modulation in our extended methods section.

Page 26, methods: “*When SNA and PNA were both present, bidirectional modulation effects between PNA and SNA (P-S and S-P) (Fig 2B) were formulated and calibrated to account for the non-additivity effects (6, 42, 80, 81) and to be consistent with experimental measurements (Fig 2A; grey dots) (38). The non-additivity effects with ISO and CCh were implicitly modeled (e.g., via cAMP-PKA dependent pathways or crosstalk between sympathetic and parasympathetic nervous systems) by introducing the ISO effects at a given concentration of CCh as follows:*

$E_{ISO}=e_{iso}\times\frac{k_{p\_s}^{h}}{k_{p\_s}^{h}+{CCh}^{h}}$*,*

*where e_iso_ is the stand-alone ISO effects as previously described (48), k_p_s_ is the P-S modulation constant, and h is the Hill coefficient. Furthermore, CCh effects with ISO were introduced as*

$E_{CCh}=e_{cch}\times k_{s\_p}$*,*

*where e_cch_ is the CCh effects alone as previously described (48), and k_s_p_ is the S-P modulation scaling factor (e.g., to account for the cross-talk between SNA and PNA (6, 42, 80, 81)). ”*

6.  I was puzzled by the data in Figure 3.  It appears that the stable pacing/firing under conditions of SNAB is actually quite broad (albeit at a low FR).  It is not clear from the text or the data in the Figure, why the blue line is drawn in the middle of the region corresponding to stable firing/pacing.  Please explain.  Is it possible that the simulations are unstable?  I find it curious that the FR displays a trough at selected values of MO and CO.  This does not seem to support conclusions related to robustness.

RE: We regret any confusion arising from an analytical error discovered in our original numerical analysis related to the SNA blockade condition. In our revised manuscript, we have rectified our implementation, separated no firing and irregular firing regions, extended our adult and aged models by considering circadian rhythms of body temperature, and reproduced Fig 3 with extended models and better labeling.

Page 15, results:

**Fig 3**. **The *alliance of ANS, CRBT and LCR* is essential to achieve circadian flexibility and performance *while preserving robustness* in SANC automaticity.** …… *(C-G) CO-MO parameter space maps at ZT6 and ZT18 under (CRBT-/-) (C), (CRBT-/-; LCR-/-) (D), (CRBT-/-; ANS-/-) (E), (CRBT-/-; SNA-/-) (F), and (CRBT-/-; PNA-/-) (G) conditions. …….*

7.  The specific changes made to the model to mimic aging need to be clearly defined and justified.  These could be interesting results but their “robustness” and relevance are unclear from the presentation of the results.  The authors conclude that “circadian amplitude of SANC in aging is mainly influenced by LCR (70%), attributable to aging-related ion channel remodeling and PNA impairment.”  The basis for this statement is unclear and difficult to reconcile with the small contribution of LCR to the circadian fluctuations.  The authors need to more clearly justify and present these results.

RE: In our original manuscript, although the circadian amplitude of FR in aged mice was significantly reduced, the relative contribution of LCR in promoting the circadian amplitude of FR became more important due to a major reduction in PNA. In the revised manuscript, our aging model has been extensively extended to incorporate a more comprehensive representation of aging-dependent changes in ion channel modeling, Ca dysfunction, CRBT disruption, and PNA impairment. As a result, our original conclusion regarding LCR in aging does not hold or becomes inaccurate with this additional level of complexity. We have now revised the manuscript accordingly to illustrate the modified role of LCR in determining SANC flexibility in aged mice.

Page 18, results: “…… *However, in aged model, different mechanisms underlying the circadian flexibility and performance of SANC were observed (Fig 5B). While the effects of ANS inhibition remain most sensitive, leading to a reduction in SANC flexibility (-77.9%) and performance (-15.9%), the effects of CRBT and LCR inhibition are enhanced, causing a reduction in flexibility (-56.6% and -31.9%, respectively) compared to the adult model (Fig 5A-5B). This enhanced effect may be attributable to aging-dependent PNA impairment. In addition, the effect of [Na]i+ clamping becomes diminished and reversed in the aged model (Fig 5B) compared to the adult model (Fig 5A).”*

Page 19, results:

**Fig 5**. **Distinct mechanisms underlying circadian patterns of SANC FR in adult and aged mice.** *(A-B) Relative contributions to SANC flexibility and performance are quantified by selective inhibition of each module in the adult (A) and aged (B) models……*

***Reviewer #3:***

“Central and peripheral clocks synergistically enhance circadian robustness, flexibility and performance of cardiac pacemaking ” by Li and Kim is an ambitious attempt to understand how 24-hour oscillations in receptor-mediated signaling and ion channel expression impact sinoatrial nodal cells' firing rate and functionality. They extend the modelization to include a model of sinoatrial node cells in aged mice. The authors are commended for their approach to model a complex interaction at a new level of detail. My high enthusiasm for the work is somewhat diminished by the lack of data showing the temperature's impact on the system. Temperature addition may be complex, but a fundamental component of intrinsic circadian signaling is the daily rhythm in core body temperature. The addition is expected to impact the results of the work and perhaps its conclusions. This limitation should be addressed. Another challenge is that the article contains a lot of jargon and abbreviations. The jargon and the numerous non-standard abbreviations lower the understandability and readability of the article. The authors are encouraged to define terms clearly. The authors are encouraged not to use many non-standard abbreviations to improve readability. Lastly, some aspects of the premise appear misrepresented or overstated.

**Concerns:**

1. Please provide a clear, succinct definition for the following terms in a table: circadian rhythms (this has many different meanings based on the field), circadian flexibility, reliance and adaptability of circadian rhythms, and circadian robustness. The authors are encouraged to use terms/definitions widely accepted by chronobiologists. This should broaden research interest in this important work.

RE: Many thanks for your comments and suggestions regarding circadian rhythms, flexibility, and robustness, and choices of terms/definitions. To address this issue and improve the clarity of our manuscript, we’ve included a new Table 1 to clearly define robustness, flexibility, and performance in the context of circadian rhythms in heart rates, and revised our manuscript accordingly. In addition, we’ve also revised Fig 3 and Fig 4, clearly labeling performance, flexibility, and robustness on each CO-MO parameter space map to improve overall readability. See our response to reviewer 1, minor comment 1 for details.

2. Please reduce the number of non-standard abbreviations to improve readability.

RE: Many thanks for your suggestions. We have significantly reduced the number of non-standard abbreviations used in our manuscript, e.g., ECG, GPCR, AC, SNAB, PNAB, ANSB, LCB, iHR, NUC, CYT, SS, CaMKII, TTFL etc., and have revised Fig 1 with a reduced number of abbreviations and a list of key term definitions. In addition, we’ve included a new Table 2 following Fig 1 to list all definitions for non-standard abbreviations (see our response to reviewer 1, comment 12).

3. Please discuss or include temperature rhythms. A hallmark feature of biological rhythms is that the 24-hour rhythm in core body temperature serves as an intrinsic Zeitgeber for local circadian clocks in the peripheral tissues. Please discuss how a daily rhythm in core body temperature may play a role in the modelization. Can the authors include this concept in the simulations?

RE: Many thanks for your suggestion regarding circadian rhythmicity in core body temperature. We have now extended our model based on experimental studies to investigate the role of body temperature in regulating cardiac pacemaking function in adult and aged mice. The experimental study by Barazi et al. 2021was performed by maintaining the core temperature at 37 °C in anesthetized mice. To incorporate circadian rhythmicity in body temperature, we introduced a simplified circadian variation in body temperature (36 °C at ZT6, 38 °C at ZT18) as documented in the literature, by introducing temperature-dependent changes in ion channel, exchanges, pumps, and autonomic balance. Our simulated results yield a Q_10_ value for SANC FR of 2, which is consistent with experimental measurements by Wu et al. 2023 and Walsh 1969. In addition, we also extended our aging model to consider aging-dependent changes in CRBT (e.g., 36 °C at ZT6, 37 °C at ZT18; Sanchez-Alavez et al. 2011). In our revision, we’ve included a new paragraph in the introduction to address CRBT. Additionally, we have revised all of our figures and the corresponding sections in our manuscript to delve into the role of CRBT in circadian rhythms of SANC function. Furthermore, we have extensively revised our methods section to provide full details of model development regarding CRBT.

Page 5, introduction: “*Besides the ANS, circadian rhythms in HR are tuned by the ups and downs of core body temperature (BT), which is regulated by the master clock in the SCN as well (Fig 1) (7, 8). BT modulates HR by inducing temperature-dependent changes in autonomic balance, intracellular ionic diffusion processes, and the properties of ion channels, exchangers, and pumps (9-14). For instance, experimental studies demonstrated that as BT increases, PNA declines with an increase in SNA (14). Furthermore, an elevation in BT can enhance SANC excitability by regulating the conductance and gating kinetics of ionic channels (12, 13), as well as the dynamics of intracellular Ca cycling (11). Consequently, as BT rises, HR accelerates with a temperature coefficient (Q10) of ~2 in rodents (14, 15). In adult mice, circadian rhythms of BT (CRBT), ranging from 36°C to 38°C, is characterized by a decline in BT during the day, followed by an increase in BT after waking up at night (16-18).”*

Page 7, introduction:

**Fig 1**. **Model schematic for *ANS, CRBT and LCR* in mouse SANC.** …… *CRBT regulates the autonomic balance between SNA and PNA, and alters the kinetics and/or conductance of ion channels, exchangers, and pumps.* ……

Page 12, results:

**Fig 2**. **Quantitative reconstruction of diverse circadian patterns in SANC FR under various conditions.** …… *(F-G) Circadian rhythmicity of BT (36~38°C) enhances the circadian amplitude of FR under control conditions (grey dots) (F) with simulated Q10=2 in agreement with experimental studies (G) (15). (H-I) In aged model, simulated maximal FR reduction (25%; red dots) (H) is consistent with experimental findings (I) (25) ……*

Page 20, discussion: “Our study sheds light on a critical yet understudied dimension of time-of-day interactions between master and local clocks in cardiac pacemaking function and dysfunction, by developing a model of *ANS, CRBT, and LCR* in SANC to recapitulate diverse circadian patterns in both adult and aged mice. Leveraging this model, we elucidated the distinctive roles of ANS, CRBT, and LCR as a dominant amplifier, a primary booster, and a secondary booster, respectively, in achieving circadian flexibility and performance with robustness in SANC. As illustrated in Fig 6A, departing uphill from the baseline state (grey circle), the introduction of LCR (green circle) moderately enhances SANC flexibility and slightly increases performance without affecting robustness. SNA addition (yellow circle) primarily enhances SANC robustness and performance, while PNA (blue circle) promotes SANC flexibility at the expense of both performance and robustness. The combination of ANS and LCR (grey circle) significantly enhances both flexibility and performance without compromising robustness. *The final incorporation of CRBT (grey dot; adult) further augments both flexibility and performance.*

However, under aging conditions, the cooperative alliance of ANS, CRBT, and LCR is disrupted, resulting in a substantial reduction in the performance and flexibility of SANC. Surprisingly, SANC robustness remains well-preserved. As shown in Fig 6B, departing downhill from the adult state (grey dot), aging-dependent ion channel remodeling (blue circle) undermines all aspects of the SANC function. However, aging-dependent PNA impairment (yellow circle) enhances both SANC performance and robustness with a trade-off in flexibility, countering the effects of MO remodeling. Moreover, aging-dependent Ca2+ handling alterations (green circle) introduce a minor reduction and enhancement in performance and flexibility respectively, without affecting robustness. The combined effects of aging-dependent changes in MO, PNA, and CO (red circle) further reduce SANC flexibility, yet fully restore robustness compared to the adult state (grey dot). *With the final addition of CRBT disruption in aging, SANC flexibility and performance are both further reduced without affecting robustness*. Comparing Fig 6A and 6B, it is intriguing to observe the well-preserved robustness evident in both adult and aged states (56, 57). In adult model, SNA counters PNA, ensuring the preservation of robustness. In aged model, the impairment of PNA works against ion channel remodeling to restore SANC robustness and maintain basal cardiac function.”

Page 26, methods: “*CRBT (Fig 1) was added and calibrated as a circadian function of BT to reproduce the circadian patterns of BT in adult mice (with averaged time-of-day BT of 37°C and a circadian amplitude of 1°C) (38) (Fig 2F; grey dots). For this, BT was modified from its original form* $BT={BT}_{a}$ *(48) as follows:*

$BT={BT}_{a}+A_{BT}\times\cos(\frac{2\pi\times(T+6)}{T_{BT}})$*,*

*where BT_a_ is the reference BT, A_BT_ is the circadian amplitude of BT, T_BT_ is the period of BT variations, and T is the current time in ZT hours.*

*To account for the effects of CRBT for all ion channels (excluding ionic pumps and exchangers) in our model, we introduced two temperature-dependent factors* $\varphi\left( BT \right)$ *and* $\eta\left( BT \right)$*, to scale gating kinetics and conductance of ion channels respectively, as previously described in (10, 45) as follows:*

$\varphi\left( BT \right)=Q_{10}^{\frac{BT-{BT}_{a}}{10}}$*,*

$\eta\left( BT \right)=1+B\left( BT-{BT}_{a} \right)$*,*

*where BT is the current body temperature, BT_a_ is the reference body temperature, B is a scaling coefficient. For example, the temperature dependence of ion channel x was modeled as follows:*

$$\tau_{x,BT}=\frac{\tau_{x}}{\varphi\left( BT \right)}$$

$G_{x,BT}=\eta\left( BT \right)G_{x}$*,*

*where G_x_ and τ_x_ are the original conductance and gating time constant of ion channel x as in the baseline model, G_x,BT_ and τ_x,BT_ are the conductance and gating time constant of ion channel x at a given BT.*

*For ionic pumps, exchangers, and intracellular ionic diffusion parameters, their temperature-dependent behaviors were modeled by scaling their maximal activity or original value (if a constant) with* $\varphi\left( BT \right)$*. For SNA and PNA, it has been reported in earlier experiments (14) that around normal BT (e.g., 37°C), as BT increases, there is a linear increase and decrease in SNA and PNA, respectively, to promote HR acceleration. Thus, the temperature-dependent behaviors of SNA and PNA were modeled by scaling their activities with* $\eta\left( BT \right)$ *based on experimental findings (14). ”*

4. Figure 2A data is based on data from Barazi. The authors show hourly data with error bars, but the original article shows around five-time points. How was the data in Figure 2A generated? The authors are encouraged to show only the actual data or alter the description of Figure 2A. It is misleading as presented.

RE: The data presented in Figure 2A was fitted (rather than raw). Employing a sine function as described in the original article by Barazi et al. 2021, we opted to use fitted data for better comparison with our model simulation results in Fig 2B. To solve this issue, we have revised our Fig 2A and its legend to avoid any confusion (see our response to reviewer 2, comment 4).

5. The Barazi article also suggests no significant fluctuation in the 24-hour rhythm in the heart rate with ANS block or PNS block (Figure 1 in Barazi). Why do the authors discuss these data sets as being circadian? What was the data that the authors used to make this conclusion?

RE: Indeed, Barazi et al. (2021) indeed show a minimal (~2%) circadian amplitude in HR variation with full ANS blockade. However, the authors also discuss potential mechanisms for this residual variation, including daily changes in SA node properties driven by the local circadian clock and alterations in HCN4 channel expression (Wang et al., 2021). This aligns with other studies showcasing residual circadian rhythmicity in HR even with ANS blockade (Gottlieb et al., 2021; Hayter et al., 2021). Recognizing the multifactorial nature of residual rhythmicity, our study specifically focused on the role of LCR in regulating IHCN channel currents, which have been shown to play a crucial role in the circadian pacemaking of the sinoatrial node (D'souza et al., 2021). Additionally, D'souza et al. demonstrated that a blockade of IHCN channels eliminated circadian rhythms in intrinsic HR, further supporting the relevance of our modeling choice. While the amplitude of the residual rhythm with ANS blockade may be small, investigating the underlying mechanisms, as our model does, contributes to a deeper understanding of circadian heart rate regulation.

Page 5, introduction: “…… However, under ANS *blockade* conditions, further studies demonstrate the significant contribution of LCR in promoting diurnal variations in the *intrinsic* HR (*HR with no autonomic control*) (22, 23). Notably, circadian rhythms in *intrinsic* HR can be eliminated with the blockade of hyperpolarization-activated cyclic nucleotide–gated channel (IHCN) (19), confirming the essential role of IHCN in mediating LCR within the sinoatrial node *(Fig 1; green dot).*”

Page 9, results: *“…… Moreover, even after a complete ANS blockade, circadian HR variations persisted (Fig 2A; green dashed line) with an amplitude of ~2% (22, 23). Although this amplitude is minimal, this suggests that in addition to the ANS and CRBT, there can be other factors or mechanisms involved in regulating the circadian rhythmicity of HR in anesthetized mice, e.g., the daily changes in the intrinsic properties of the sinoatrial node arising from changes in the expression of IHCN (Fig 1; green dot) (19, 38).”*

6. ANS block data in Figures 2A and 2B are qualitatively different when the amplitude peaks (ZT 24 vs ZT 18). Why?

RE: In our original manuscript, there was a phase difference between experimental (peaked at ZT24) and simulated (peaked at ZT18) ANS block data. This discrepancy arose because we modeled the trough of LCR to be aligned at ZT6 (with a smaller I_HCN_ current) based on D’souza et al., Heart Rhythm, 2021. However, without ANS, circadian rhythms of HR showed troughs at ZT12 as reported by Barazi et al. We speculated that LCR could be phase-shifted due to the lack of external cues from ANS. To address this issue and prevent confusion, we have now revised our simulation and Fig 2 to adjust the phase of LCR according to the presence of ANS, so that without ANS, LCR shows troughs at ZT12, and with ANS (either PNA or SNA), LCR shows troughs at ZT6. To address this issue, we also revised Fig 2 (see our response to reviewer 2, comment 4) and included a more detailed and updated description in the methods section regarding LCR and full ANS block data.

Page 24, methods: “*First, without considering CRBT, LCR was introduced and calibrated into the baseline model as a circadian variation in the I_HCN_ (Fig 1; green dot) to generate a weak circadian amplitude (~2%) in SANC FR as observed in adult mice under full ANS blockade with BT=37°C (Fig 2A; green dashed line) (19, 38). Specifically, to introduce circadian variations in I_HCN_ into the baseline model with ANS blockade, I_HCN_ was modified from its original form*$I_{HCN}=I_{HCN,Na}+ I_{HCN,K}$ *as follows:*

$I_{HCN}=\left( 1+ A_{LCR}\times cos (\frac{2\pi T}{T_{LCR}}) \right)\times\left( I_{HCN,Na}+ I_{HCN,K} \right)$*,*

*where A_LCR_ is the amplitude of LCR, T_LCR_ is the period of LCR, T is the current ZT in hours,* $I_{HCN,Na} \text{and} I_{HCN,K}$ *are the Na^+^ and K^+^ specific HCN channel currents, respectively (48). It should be noted that, while experimental recordings of HR with ANS blockade (38) show troughs at ZT12, experimental measurements of I_HCN_ currents (19) show troughs at ZT6. We speculated that this might be attributable to a phase shift in LCR due to a lack of external cues from the ANS. For this, when the ANS is present, I_HCN_ was modeled as follows:*

$I_{HCN}=\left( 1+ A_{\text{LCR}}\times cos (\frac{2\pi\times(T+6)}{T_{LCR}}) \right)\times\left( I_{HCN,Na}+ I_{HCN,K} \right).$”

**Reviewer #4:**

This paper is focused on circadian regulation of cardiac excitability, an important aspect of physiology that has not been thoroughly addressed in previous computational modeling studies. The authors extend a published model of action potential generation in mouse sinoatrial node cells (SANC) to incorporate the regulation of cardiac pacemaking by both the central circadian clock—through the autonomic nervous system--and local circadian rhythmicity (LCR) within the heart. The authors perform simulations and sensitivity analysis to assess the role of parasympathetic nervous activity (PNA), sympathetic nervous activity (SNA), and LCR on circadian rhythms in heart rate (HR). They also use their model to study mechanisms underlying the age-related decline of circadian rhythms in HR.

Overall, the study is well-motivated, and the manuscript is well-written. The findings on the importance of non-additive interactions between PNA and SNA for reproducing circadian patterns in SANC firing rate are potentially interesting. My main concern is that the evidence supporting this finding is not yet adequately explained in the manuscript, as I describe in my comments below.

***Major comments***

1. It is stated on lines 175-178 that when additive PNA and SNA (no PNA-SNA interactions) is assumed, the baseline of the simulated circadian rhythms is approximately 5% lower than the experimental data. I don’t understand how this 5% is calculated… for example, it looks like the trough of the simulated curve is 400 BPM at ZT6 (Fig 2B, grey boxes) compared to a trough of 500 BPM in the experimental data at ZT6 (Fig 2A, grey dots), which would correspond to a 20% difference. A similar calculation using the peak of the simulated and experimental curves at ZT18 (475 and 575 BPM, respectively) gives a 21% difference. How was the 5% result obtained?

RE: We apologize for any confusion surrounding Fig 2A. In our original manuscript, we compared normalized data between experimental measurements and model simulations (normalized to the average time-of-day HR with ANS blockade), rather than using the raw data in BPM. When normalized (to HR/FR at ZT6 with ANS blockade), our model simulation assuming additive SAN and PNA exhibited a ~5% deviation from the experimental data. To solve this issue, we have now revised Fig 2 and its legend, and our manuscript, accordingly.

Page 10: “…… However, *in the absence of the PNA-SNA interactions (i.e., additive PNA and SNA), the baseline of the simulated circadian rhythm of normalized FR (Fig 2B; red box) was lower by ~5% in comparison to the experimental data (Fig 2A; grey dashed line)*……”

Page 12, results:

**Fig 2**. **Quantitative reconstruction of diverse circadian patterns in SANC FR under various conditions.** (A) Averaged circadian HR fluctuations in anesthetized mice (BT=37°C) with 12-h light cycles before (control; grey dashed line) and after SNA blockade (blue dashed line), PNA blockade (yellow dashed line), and ANS blockade (green dashed line) (38). *Circadian fluctuations were fitted using a sine function as described in (38) and normalized to HR at ZT6 with ANS blockade*. (B) Simulated circadian FR patterns in a single mouse SANC (BT=37°C; *normalized to FR at ZT6 with ANS blockade*) closely recapitulate experimental findings (38). (C-D) Model simulations accurately reproduce minimal and maximal time-of-day FR (C), and normalized circadian amplitudes of FR (D) under control, SNA blockade, PNA blockade, and ANS blockade conditions. The normalized amplitude values are obtained by dividing circadian FR amplitudes in beats per minute (BPM) by the mean time-of-day FR. (E) *Either additive or unidirectional modulation effects alone are insufficient to accurately reproduce time-of-day FRs under the control conditions* ……

2. The authors go on to say that when they implemented bidirectional modulation effects, the circadian pattern in FR under the control condition was accurately simulated (Fig 2B, grey line). The trough and peak of this simulated curve appear to be at 425 and 500 BPM at ZT6 and ZT18, which are still 17% and 15% lower than the corresponding experimental values, respectively. Thus, more explanation is needed to justify the claim of accurate simulation as well as the statement on line 199-200 that the incorporation of bidirectional effects “aligns the simulated FR with experimental data (A; grey dots)”.

RE: We apologize again for any confusion regarding Figs 2A and 2B. In Barazi et al. 2021, heart rates were estimated from telemetry ECG recordings; however, our model simulation of firing rates was performed using a single sinoatrial nodal (SAN) cell model. Due to tissue-level complexities, e.g., intercellular coupling, electrophysiological heterogeneity, and SAN-atrial tissue coupling, we consider heart rate as an organ-level property emergent from the collective behaviors of many coupled SAN cells, and firing rate as a cellular property of single SAN cells. As reported by Larson et al., PNAS, 2013, heart rates could be up to ~30% higher than firing rates measured in single SAN cells. Thus, we chose to compare normalized experimental data of heart rates and model simulation of firing rates to reconstruct various circadian patterns presented in Fig 2. To address this issue, we have revised Fig 2 and its legend accordingly (see our response to reviewer 2, comment 4).

3. Based on the above calculations, the simulated non-additive curve (grey line) does indeed agree better with the experimental curve (grey dots) than the simulated additive curve (grey boxes) by a few percent, but given the large error bars associated with the grey dots in the experimental data (Fig 2A) it is not clear to me that there is enough evidence to support the strong claim on lines 186-187 that the “results unequivocally indicate that the non-additivity of PNA and SNA is required to properly construct the circadian patterns of SANC function”. Is it not possible that with other parameter choices, the experimental data could be equally well-recapitulated with purely additive effects?

RE: We concur with the reviewer's observation that our simulated non-additive curve outperforms the additive curve by a few percentage points, despite the notable error bars associated with the experimental data. Our modeling approach aims to quantitatively replicate mean average measurements in experimental findings (Fig 2D) (see our response to comment 1). With only additive effects, reconstructing circadian patterns of heart rate (HR) under control conditions (Fig 2A; dashed line) would be challenging. Model parameterization for sympathetic (SNA) and parasympathetic (PNA) activity is well-constrained under PNA blockade and SNA blockade conditions, respectively. However, when calibrating the model under control conditions (with combined SNA and PNA), the only remaining degree of freedom is how these branches interact. Without considering these interactions, options for model calibration become limited. Moreover, the non-additive interactions between the parasympathetic and sympathetic branches of the autonomic nervous system (ANS), well-documented as "accentuated antagonism" since the 1970s (Levy 1971, Levy 1977, Levy and Schwartz 2004, Dawson et al. 2008, Coote 2013) further support our modeling choice. To address this concern, we have revised the manuscript, incorporating additional references on parasympathetic and sympathetic interactions, adjusting the figure accordingly (see our response to comment 1), and providing a detailed description of our model development in this context.

Page 10, results: “…… This suggests that the non-additivity of PNA and SNA is required to properly reconstruct the circadian patterns of SANC function. *This finding is in agreement with the interactions between the sympathetic and parasympathetic branches of the ANS as accentuated antagonism (42-44) ……”*

Page 26, methods: “*When SNA and PNA were both present, bidirectional modulation effects between PNA and SNA (P-S and S-P) (Fig 2B) were formulated and calibrated to account for the non-additivity effects (6, 42, 80, 81) and to be consistent with experimental measurements (Fig 2A; grey dots) (38). The non-additivity effects with ISO and CCh were implicitly modeled (e.g., via cAMP-PKA dependent pathways or crosstalk between sympathetic and parasympathetic nervous systems) by introducing the ISO effects at a given concentration of CCh as follows:*

$E_{ISO}=e_{iso}\times\frac{k_{p\_s}^{h}}{k_{p\_s}^{h}+{CCh}^{h}}$*,*

*where e_iso_ is the stand-alone ISO effects as previously described (48), k_p_s_ is the P-S modulation constant, and h is the Hill coefficient. Furthermore, CCh effects with ISO were introduced as*

$E_{CCh}=e_{cch}\times k_{s\_p}$*,*

*where e_cch_ is the CCh effects alone as previously described (48), and k_s_p_ is the S-P modulation scaling factor (e.g., to account for the cross-talk between SNA and PNA (6, 42, 80, 81)).* ”

4. The authors write on lines 183-184 that both minimal and maximal time-of-day FR are in quantitative agreement with the experimental data (Fig 2C). The caption of Fig 2C explains that the FR values were normalized to FR at ZT12 under the ANSB condition. I’m afraid I don’t quite understand how this normalization was applied. The curve of green dots in Fig 2A appears to reach a minimum at ZT12, thus I would expect the Normalized HR Min value under the ANSB condition to be exactly 1 for the experimental data. However, in Fig 2C the Normalized HR Min value under the ANSB condition shown for the experimental data is less than 1.

RE: We apologize for any confusion regarding the normalization in Fig 2. We normalized both experimental and model simulation data to the average (or ZT6) time-of-day heart/firing rates with ANS blockade, and that’s why the normalized HR min value is less than 1. To clarify this matter, we have revised Fig 2 and its legend accordingly (see our response to comment 1).

5. Why does the simulated FR curve under ANSB condition (Fig 2B green line) have a trough at ZT6, whereas the experimental FR curve under ANSB condition (Fig 2A green dots) has a trough at ZT12? Does this discrepancy affect the appropriateness of using FR at ZT12 under ANSB condition as the reference value for normalization?

RE: In our original manuscript, there was a phase difference between experimental (peaked at ZT24) and simulated (peaked at ZT18) ANS block data. This was because we modeled the trough of LCR to be aligned at ZT6 (with a smaller I_HCN_ current) based on D’souza et al., Heart Rhythm, 2021. However, without ANS, circadian rhythms of HR showed troughs at ZT12 as reported by Barazi et al. We speculated that LCR could be phase-shifted due to the lack of external cues from ANS. To address this issue and avoid confusion, we have now revised our simulation and Fig 2 to adjust the phase of LCR according to the presence of ANS, so that without ANS, LCR shows troughs at ZT12, with ANS (either PNA or SNA), and LCR shows troughs at ZT6. To address this issue, we also revised Fig 2 (see our response to reviewer 2, comment 4) and included a more detailed and updated description in the methods section regarding LCR and full ANS block data (see our response to reviewer 3, comment 6).

6. In general, why are the simulated FR lower than the experimental HR, as evidenced by the lower range used for the y-axis in Fig 2B (300 to 550 BPM) than Fig 2A (350 to 700 BPM)? It is not necessary for a model to exactly reproduce the same range of values as seen in experiments to be useful, but since the simulated FRs are systematically and significantly lower than the experimental HRs it would be helpful for the authors to explain or at least comment on this discrepancy. Furthermore, this difference between the simulated FR and the experimental HR suggests that it might be more appropriate to describe the results as “qualitative” rather than “quantitative” reconstructions of diverse circadian patterns in SANC FR the section heading on line 164 and the Fig 2 caption. Similarly, is it accurate to say on line 173 that the model simulations “meticulously recapitulated” the experimental findings given that there is about a 100 BPM difference between the two?

RE: In Barazi et al. 2021, heart rates were estimated from telemetry ECG recordings; however, our model simulation of firing rates was performed using a single sinoatrial nodal (SAN) cell model. Due to tissue-level complexities, e.g., intercellular coupling, electrophysiological heterogeneity, and SAN-atrial tissue coupling, we consider heart rate as an organ-level property emergent from the collective behaviors of many coupled SAN cells, and firing rate as a cellular property of single SAN cells. As reported by Larson et al., PNAS, 2013, heart rates could be up to ~30% higher than firing rates measured in single SAN cells. Thus, we chose to compare normalized experimental data of heart rates and model simulation of firing rates to reconstruct various circadian patterns presented in Fig 2. Without clarifying these details, we concur with the reviewer that the assertion claiming our model “meticulously recapitulated” the experimental data could be confusing. To address this issue, we have revised Fig 2 and its legend accordingly (see our response to reviewer 2, comment 4).

7. More details on how the model was calibrated to experimental data should be provided. For example, on lines 55-57 the authors say the bidirectional modulation effects P-S and S-P were formulated and calibrated to account for the non-additivity effects (8-11) but it is not explained what these effects are. Similarly, it is mentioned that the model was calibrated for time-of-day PNA (line 420), time-of-day SNA (line 423), age-dependent MO reduction (line 436), aging-dependent PNA impairment (line 439), but it is not described how exactly the model was calibrated. Were the data sufficient to constrain the parameters, or are there multiple parameter sets that can reproduce the data well? Some discussion of parameter identifiability and how the authors modeling approach and parameter fitting does or does not account for the variability/heterogeneity of cardiac electrophysiology in general (and more specifically, the variability/heterogeneity depicted by the error bars in Fig 2A) seems warranted.

RE: To address this issue, we’ve now extensively extended our methods section with full details of model development, calibration, and validation, and added a new Table S1 (Supporting Information) to list all parameter definitions and values with their sources cited.

Page 24, methods*: “Autonomic, electrophysiologic, and circadian properties of cardiac function are species-dependent (26, 79). Given the availability of experimental data in mice, our model is constructed to be mouse-specific. We used the mathematical model of mouse SANC AP developed by Ding et al. (48, 51), based on the original work of Kharche et al. (75), as our baseline model. All baseline model definitions, equations, and parameter settings were unchanged from those previously described and implemented by Ding et al. (48). Due to the absence of circadian regulation in the baseline model, we extended it to quantitatively describe circadian variations in ANS, CRBT, and LCR for both adult and aged mice. Detailed descriptions of model equations are provided in the following section, along with all parameter values and settings listed in Table S1 (Supporting Information).*

*Incorporation of LCR into the baseline model*

*First, without considering CRBT, LCR was introduced and calibrated into the baseline model as a circadian variation in the I_HCN_ (Fig 1; green dot) to generate a weak circadian amplitude (~2%) in SANC FR as observed in adult mice under full ANS blockade with BT=37°C (Fig 2A; green dashed line) (19, 38). Specifically, to introduce circadian variations in I_HCN_ into the baseline model with ANS blockade, I_HCN_ was modified from its original form*$I_{HCN}=I_{HCN,Na}+ I_{HCN,K}$ *as follows:*

$I_{HCN}=\left( 1+ A_{LCR}\times cos (\frac{2\pi T}{T_{LCR}}) \right)\times\left( I_{HCN,Na}+ I_{HCN,K} \right)$*,*

*where A_LCR_ is the amplitude of LCR, T_LCR_ is the period of LCR, T is the current ZT in hours,* $I_{HCN,Na} \text{and} I_{HCN,K}$ *are the Na^+^ and K^+^ specific HCN channel currents, respectively (48). It should be noted that, while experimental recordings of HR with ANS blockade (38) show troughs at ZT12, experimental measurements of I_HCN_ currents (19) show troughs at ZT6. We speculated that this might be attributable to a phase shift in LCR due to a lack of external cues from the ANS. For this, when the ANS is present, I_HCN_ was modeled as follows:*

$$I_{HCN}=\left( 1+ A_{\text{LCR}}\times cos (\frac{2\pi\times(T+6)}{T_{LCR}}) \right)\times\left( I_{HCN,Na}+ I_{HCN,K} \right).$$

*Incorporation of the circadian regulation of ANS into the baseline model*

*Then, circadian PNA (Fig 1; blue dot) was added and calibrated as a circadian function of carbachol (CCh) concentrations, to reproduce the circadian amplitude (~7%) effects based on experimental data under SNA blockade conditions with BT=37°C (Fig 2A; blue dashed line) (38). For this, CCh was modified from its original form* $CCh={CCh}_{basal}$ *(48) as follows:*

$CCh={CCh}_{basal}+A_{CCh}\times\cos(\frac{2\pi\times(T-6)}{T_{CCh}})$*,*

*where CCh_basal_ is the basal concentration of CCh, A_CCh_ is the circadian amplitude of CCh, T_CCh_ is the period of CCh variations, and T is the current time in ZT hours. This circadian variation in CCh leads to the circadian variation in the muscarinic K^+^ current (I_KACh_) because I_KACh_ is regulated by CCh in the original baseline model as follows (Fig 1; blue dot) (48):*

$I_{KACh}=G_{KAch}\times w\left( V,CCh,t \right)\times(V-E_{K})$*,*

*where G_KACh_ is the maximal conductance of I_KACh_, V is the membrane potential in mV, t is current time in ms, w(V,CCh,t) is the voltage- and CCh-dependent channel activation function, and E_K_ is the K^+^ equilibrium potential in mV.*

*Furthermore, SNA (Fig 1; yellow dot) was implemented to mimic isoproterenol (ISO) effects by modifying the kinetics and/or conductance of I_st_, I_Na1.1_, I_CaT_, I_CaL_, I_K1_, I_Ks_, I_Kr_, I_to_, and the activities of J_Rel_ and J_up_, using parameter settings as previously described (see Supporting Information Table S1 for more details) (48). The administration of ISO was scaled to align with experimental measurements of HR acceleration under PNA blockade conditions (BT=37°C) (Fig 2A; yellow dots) (38). A combination of LCR and ISO administration is enough to generate a circadian amplitude (~3%) in SANC FR as measured in the experiments (38), allowing little room for additional in-phase circadian variations in SNA. Therefore, SNA in this study was modeled with no circadian variations when CRBT is not present (BT=37°C). This modeling choice aligns with earlier experimental findings indicating a high sympathetic drive in mice, potentially limiting additional time-of-day variations to maintain a normal core temperature (37°C) under standard laboratory conditions (20°C) (26, 74).*

*When SNA and PNA were both present, bidirectional modulation effects between PNA and SNA (P-S and S-P) (Fig 2B) were formulated and calibrated to account for the non-additivity effects (6, 42, 80, 81) and to be consistent with experimental measurements (Fig 2A; grey dots) (38). The non-additivity effects with ISO and CCh were implicitly modeled (e.g., via cAMP-PKA dependent pathways or crosstalk between sympathetic and parasympathetic nervous systems) by introducing the ISO effects at a given concentration of CCh as follows:*

$E_{ISO}=e_{iso}\times\frac{k_{p\_s}^{h}}{k_{p\_s}^{h}+{CCh}^{h}}$*,*

*where e_iso_ is the stand-alone ISO effects as previously described (48), k_p_s_ is the P-S modulation constant, and h is the Hill coefficient. Furthermore, CCh effects with ISO were introduced as*

$E_{CCh}=e_{cch}\times k_{s\_p}$*,*

*where e_cch_ is the CCh effects alone as previously described (48), and k_s_p_ is the S-P modulation scaling factor (e.g., to account for the cross-talk between SNA and PNA (6, 42, 80, 81)).*

*Incorporation of CRBT into the baseline model*

*CRBT (Fig 1) was added and calibrated as a circadian function of BT to reproduce the circadian patterns of BT in adult mice (with averaged time-of-day BT of 37°C and a circadian amplitude of 1°C) (38) (Fig 2F; grey dots). For this, BT was modified from its original form* $BT={BT}_{a}$ *(48) as follows:*

$BT={BT}_{a}+A_{BT}\times\cos(\frac{2\pi\times(T+6)}{T_{BT}})$*,*

*where BT_a_ is the reference BT, A_BT_ is the circadian amplitude of BT, T_BT_ is the period of BT variations, and T is the current time in ZT hours.*

*To account for the effects of CRBT for all ion channels (excluding ionic pumps and exchangers) in our model, we introduced two temperature-dependent factors* $\varphi\left( BT \right)$ *and* $\eta\left( BT \right)$*, to scale gating kinetics and conductance of ion channels respectively, as previously described in (10, 45) as follows:*

$\varphi\left( BT \right)=Q_{10}^{\frac{BT-{BT}_{a}}{10}}$*,*

$\eta\left( BT \right)=1+B\left( BT-{BT}_{a} \right)$*,*

*where BT is the current body temperature, BT_a_ is the reference body temperature, B is a scaling coefficient. For example, the temperature dependence of ion channel x was modeled as follows:*

$$\tau_{x,BT}=\frac{\tau_{x}}{\varphi\left( BT \right)}$$

$G_{x,BT}=\eta\left( BT \right)G_{x}$*,*

*where G_x_ and τ_x_ are the original conductance and gating time constant of ion channel x as in the baseline model, G_x,BT_ and τ_x,BT_ are the conductance and gating time constant of ion channel x at a given BT.*

*For ionic pumps, exchangers, and intracellular ionic diffusion parameters, their temperature-dependent behaviors were modeled by scaling their maximal activity or original value (if a constant) with* $\varphi\left( BT \right)$*. For SNA and PNA, it has been reported in earlier experiments (14) that around normal BT (e.g., 37°C), as BT increases, there is a linear increase and decrease in SNA and PNA, respectively, to promote HR acceleration. Thus, the temperature-dependent behaviors of SNA and PNA were modeled by scaling their activities with* $\eta\left( BT \right)$ *based on experimental findings (14).*

*Modeling aging-dependent alternations*

*Aging-dependent ion channel remodeling (Fig 1; red dot) was calibrated to be consistent with electrophysiological measurements by Larson et al. (25), Tellez et al. (39) and Liu et al. (40) as follows:*

$I_{CaT,aging}=a_{cat}\times I_{CaT}$*,*

$I_{CaL,aging}=a_{cal}\times I_{CaL}$*,*

$I_{HCN,aging}=a_{hcn}\times I_{HCN}$*,*

$I_{NaK,aging}=a_{nak}\times I_{NaK}$*,*

$I_{NCX,aging}=a_{ncx}\times I_{NCX}$*,*

$I_{Kr,aging}=a_{hcn}\times I_{Kr}$*,*

$$I_{Ks,aging}=a_{hcn}\times I_{Ks}$$

*where a_cat_, a_cal_, a_hcn_, a_nak_, a_ncx_, a_kr_, a_ks_ are the scaling factors for I_CaT_, I_CaL_, I_HCN_, I_NaK_, I_NCX_, I_Kr_ , and I_Ks_ under aging conditions, respectively. Moreover, the aging-dependent shift of the activation midpoint of I_HCN_ was modeled as the following:*

$V_{HCN, aging}=V_{HCN}+v_{hcn}$*,*

*where V_HCN_ is the activation midpoint of I_HCN_ in adult model, and v_hcn_ is the activation shift of I_HCN_ in aged model.*

*Aging-dependent Ca^2+^ cycling dysfunction (Fig 1; red dot) in SERCA (J_up_) and Ryanodine receptor Ca release (J_Rel_) were calibrated according to previous experimental measurements by Liu et al. (40) as follows:*

$$J_{up,aging}=a_{up}\times J_{up}$$

$$J_{Rel,aging}=a_{rel}\times J_{Rel}$$

*where a_up_, a_rel_ are the scaling factors for J_up_, J_Rel_ under aging conditions, respectively. Moreover, the aging-dependent increase in the ratio between phospholamban and SERCA (40) was modeled by an increase to the original value of cytoplasmic Ca^2+^ sensitivity coefficient (K_mf_) in the baseline model (48) as follows:*

$$K_{mf,aging}=a_{kmf}\times K_{mf}$$

*where a_kmf_ is the scaling factor for K_mf_ under aging conditions.*

*In addition, aging-related PNA impairment (Fig 1; red dot) was introduced by a major reduction in PNA to be consistent with previous experimental studies (41) as follows:*

$${CCh}_{aging}=a_{cch}\times CCh$$

*where a_cch_ is the scaling factor of CCh under aging conditions. SNA was assumed to be unchanged in aged mice as reported by Larson et al. (25).*

*Moreover, aging-dependent CRBT disruption was modeled by a reduction in both averaged time-of-day BT and circadian amplitude of BT in aged mice as previously reported (17). Specifically:*

${BT}_{aging}={BT}_{a,aging}+A_{BT,aging}\times\cos(\frac{2\pi\times(T+6)}{T_{BT}})$*,*

*where BT_a,aging_ is the reference BT in aged model, and A_BT,aging_ is the circadian amplitude of BT in aged model.”*

Supporting Information:

*Table S1. Model Parameters and Settings*

| **Parameter** |  | **Definition** | **Value/setting (reference)** | **Unit** |
| --- | --- | --- | --- | --- |
| A_LCR_ |  | The circadian amplitude of LCR | 0.25(1) | */* |
| T_LCR_ |  | The circadian period of LCR | 24(1) | *hour* |
| CCh_basal_ |  | Basal CCh concentration | 265(2, 3) | *nM* |
| A_CCh_ |  | The circadian amplitude of CCh | 85(3) | *nM* |
| T_CCh_ |  | The circadian period of CCh | 24(3) | *hour* |
| a_cch_ |  | Scaling factor for CCh in aging | 0.25(4) | */* |
| k_p_s_ |  | The P-S modulation constant | 450(3, 5) | *nM* |
| h |  | Hill coefficient | 6(3, 5, 6) | */* |
| k_s_p_ |  | The S-P modulation scaling factor | 0.5(3, 6-8) | */* |
| BT_a_ |  | The reference BT | 37(9, 10) | *°C* |
| A_BT_ |  | The circadian amplitude of BT | 1(9, 10) | *°C* |
| T_BT_ |  | The period of BT variations | 24(9, 10) | *hour* |
| BT_a,aging_ |  | The reference BT in aged mice | 36.5(10) | *°C* |
| A_BT, aging_ |  | The circadian amplitude of BT in aged mice | 0.5(10) | *°C* |
| a_cat_ |  | Scaling factor for I_CaT_ in aging | 0.46(11) | */* |
| a_cal_ |  | Scaling factor for I_CaL_ in aging | 0.69(11) | */* |
| a_hcn_ |  | Scaling factor for I_HCN_ in aging | 0.48(11) | */* |
| v_hcn_ |  | Activation shift of I_HCN_ in aging | 12(11) | *mV* |
| a_nak_ |  | Scaling factor for I_NaK_ in aging | 1.5(12) | */* |
| a_ncx_ |  | Scaling factor for I_NCX_ in aging | 0.5(13) | */* |
| a_kr_ |  | Scaling factor for I_Kr_ in aging | 0.92(12) | */* |
| a_ks_ |  | Scaling factor for I_Ks_ in aging | 1.6(12) | */* |
| a_up_ |  | Scaling factor for J_up_ in aging | 0.7(13) | */* |
| a_rel_ |  | Scaling factor for J_Rel_ in aging | 0.2(13) | */* |
| a_kmf_ |  | Scaling factor for K_mf_ in aging | 1.25(13) | */* |
| B |  | Scaling factor for the effects of CRBT on ion channels | 0.008(14, 15) | *K^-1^* |
| B_SNA_ |  | Scaling factor for the effects of CRBT on SNA | 0.096(16) | *K^-1^* |
| B_PNA_ |  | Scaling factor for the effects of CRBT on PNA | -0.04(16) | *K^-1^* |
| Q_10_ |  | The temperature coefficient | *2.18 for I_to_ and I_sus_ (17, 18)*  *1.7 for I_Na_ (17)*  *2.3 for I_CaL_(17)*  *1.5 for I_CaT_ (17)*  *1.5 for I_Kr_, I_Ks_ and I_KACh_ (15)*  *2.0 for I_HCN_ (18, 19)*  *2.6 for J_up_ (20)*  *1.6 for I_NCX_ (20, 21)*  *2.1 for I_NaK_ (22)*  *1.25 for J_Rel_ (23)*  *1.3 for J_diff_ and J_tr_ (23)*  *1.6 for Ca^2+^ buffers (24)* | */* |
| e_cch_ |  | Standalone effects of CCh | I_KACh_ activation (2) | |
| e_iso_ |  | Standalone effects of ISO | *15.9% increase in G_st_ (2, 3, 25)* | |
|  |  |  | *15.9% increase in G_Na1.1_ (2, 3, 25)* | |
|  |  |  | *85.5% increase in G_CaT_ (2, 3, 25)* | |
|  |  |  | *112% increase in G_CaL_ (2, 3, 25)* | |
|  |  |  | *15.9% increase in G_K1_ (2, 3, 25)* | |
|  |  |  | *15.9% increase in G_Ks_ (2, 3, 25)* | |
|  |  |  | *15.9% increase in G_to_ (2, 3, 25)* | |
|  |  |  | *112% increase in V_Rel_ (2, 3, 25)* | |
|  |  |  | *52.83% decrease in k_up_ (2, 3, 25)* | |
|  |  |  | *Altered steady-state activation (V_HCN_ = 87.618mV) and gating properties in I_HCN_ (2, 3, 25)* | |
|  |  |  | *16.6% increase in G_Kr_ and 5.3 mV negative shift of activation (2, 3, 25)* | |

8. Is there a way to experimentally test the model prediction that non-additive interactions between PNA and SNA are important for the circadian patterns, or to experimentally validate that these interactions were modeled appropriately? If so, it would be helpful to discuss these possibilities in the Discussion section.

RE: Yes, and we think there should be experimental protocols available to test the non-additivity and even provide additional information to better constrain model development. As illustrated in Fig 10, Behar et al. Front. Physiol, 2016, one relevant protocol could be to examine firing rate alternations in responses to ISO only, CCh only, or combined (with a difference in timing between the application of ISO and CCh). To address this issue, we have revised our manuscript accordingly.

Page 23, discussion: “*We have made several assumptions for simplicity in developing our model that may impact the generalizability and interpretation of our model simulations.……*. *Additional model development or experimental studies* would be helpful to further advance our understanding of circadian SANC function and dysfunction, e.g., *non-additive interactions between SNA and PNA*(6), the mechanistic coupling between the ANS and LCR (76), trajectory of functional aging (77), and entrainment dynamics between the master circadian clock and LCR during jet lag or with shift work disorder (78).”

**Minor Comments**

1. The authors conclude on lines 156-158 that SNA serves as a SANC robustness and performance enhancer, PNA acts as a flexibility amplifier, and LCR functions as a flexibility and performance booster. Do the words “enhancer, amplifier, and booster” as used here all mean the same thing, or were the 3 different words employed to indicate some differences in the roles that SNA, PNA, and LCR play when it comes to interpreting their simulation results? For example, would it be just as accurate to say that PNA acts as a flexibility booster or enhancer instead of as a flexibility amplifier? If so, then the authors should just pick one of these 3 words to use throughout this sentence (as well as in lines 346-349) so that different meanings aren’t implied.

RE: We agree with you that these terms could be misleading. Given that we have extended our model and simulation results extensively, we have revised our manuscript accordingly to improve readability.

Page 7, introduction: “In this study, we present a novel mathematical model that captures the intricate regulation of SANC by *ANS, CRBT, and LCR in mice (Fig 1) (Table 2).* The model accurately reproduces diverse circadian patterns as shown in previous experimental studies in adult and aged mice (19, 25, 38). Utilizing the model, *we elucidate the specific roles of ANS, CRBT, and LCR in attaining circadian flexibility, performance, and robustness in SANC*. Additionally, we quantitatively dissect SANC dysfunction during the aging process. *Our findings reveal that ANS plays a pivotal role in promoting SANC flexibility and performance, with CRBT and LCR acting as primary and secondary boosters, respectively, to further enhance SANC flexibility and performance.* However, during the aging process, while SANC flexibility and performance experience significant reductions, robustness remains mostly preserved. Specifically, the aging-related decline in PNA acts to restore SANC robustness while compromising flexibility, suggesting a potential trade-off strategy in the aging process. Our model simulations highlight a critical dimension for time-of-day interactions between ANS, CRBT, and LCR in cardiac pacemaking function and dysfunction.”

Page 20, discussion: “Our study sheds light on a critical yet understudied dimension of time-of-day interactions between master and local clocks in cardiac pacemaking function and dysfunction, by developing a model of *ANS, CRBT, and LCR* in SANC to recapitulate diverse circadian patterns in both adult and aged mice. *Leveraging this model, we elucidated the distinctive roles of ANS, CRBT, and LCR as a dominant amplifier, a primary booster, and a secondary booster, respectively, in achieving circadian flexibility and performance with robustness in SANC*. As illustrated in Fig 6A, departing uphill from the baseline state (grey circle), the introduction of LCR (green circle) moderately enhances SANC flexibility and slightly increases performance without affecting robustness. SNA addition (yellow circle) primarily enhances SANC robustness and performance, while PNA (blue circle) promotes SANC flexibility at the expense of both performance and robustness. The combination of ANS and LCR (grey circle) significantly enhances both flexibility and performance without compromising robustness. The final incorporation of CRBT (grey dot; adult) further augments both flexibility and performance.”

2. The phrase “SNA in this study was modeled as a high ISO tone” on lines 52-52 struck me as strange as I do not usually see the word tone associated with a drug. Also, a brief explanation of why using isoproterenol to model SNA would be helpful even if it was previously described in (3).

RE: To address this issue, we have revised the methods section to avoid confusion.

Page 25, methods: “*Furthermore, SNA (Fig 1; yellow dot) was implemented to mimic isoproterenol (ISO) effects by modifying the kinetics and/or conductance of Ist, INa1.1, ICaT, ICaL, IK1, IKs, IKr, Ito, and the activities of JRel and Jup, using parameter settings as previously described (see Supporting Information Table S1 for more details) (48). The administration of ISO was scaled to align with experimental measurements of HR acceleration under PNA blockade conditions (BT=37°C) (Fig 2A; yellow dots) (38). A combination of LCR and ISO administration is enough to generate a circadian amplitude (~3%) in SANC FR as measured in the experiments (38), allowing little room for additional in-phase circadian variations in SNA. Therefore, SNA in this study was modeled with no circadian variations when CRBT is not present (BT=37°C). This modeling choice aligns with earlier experimental findings indicating a high sympathetic drive in mice, potentially limiting additional time-of-day variations to maintain a normal core temperature (37°C) under standard laboratory conditions (20°C) (26, 74).*”

3. In line 144 of the Abstract, replace “Our work shed light” with “Our work sheds light”

RE: We have revised our manuscript accordingly.

Page 2, abstract: “…… Our work *sheds* light on the critical alliance of ANS, CRBT, and LCR in regulating time-of-day cardiac pacemaking function and dysfunction, offering insights into novel therapeutic targets for the prevention and treatment of cardiac arrhythmias.”

4. An easier-to-follow explanation of what simulations were performed to obtain the curves in Fig 2B would be helpful for readers to be able to reproduce the results. All of the information may already be provided in the Materials and Methods or Supporting Information, but it is not explicitly spelled out. For example, to go from the Control curve to the P-S curve, does one simply set the parameter k_{s_p} to 1? Similarly, what parameters would one change to go from the Control curve to the S-P curve, the Additive curve, or the 3 blockade curves? If the authors would prefer not to include that type of description in the manuscript itself, it could go in the readme file associated with the model code instead.

RE: To address this issue, we’ve now provided a step-by-step explanation regarding our model development, validation, and simulation protocols in the extended methods section (see our response to comment 7).
